# From metabolic fingerprints to field solutions: engineering the apple rhizosphere microbiome via host-directed *Bacillus* recruitment for sustainable apple replant disease control

**DOI:** 10.1186/s40168-025-02301-9

**Published:** 2025-12-23

**Authors:** Weitao Jiang, Ran Chen, Lefen Song, Lei Qin, Xin Xu, Xiaoxuan Li, Lei Zhao, Jinhui Lyu, Xiaoqi Wang, Gongshuai Wang, Xuesen Chen, Yusong Liu, Mei Wang, Chengmiao Yin, Yanfang Wang, Zhiquan Mao

**Affiliations:** 1 College of Horticulture Science and Engineering, Shandong Agricultural University Tai’an, Shandong, 271018 China; 2 College of Chemistry and Material Science, Shandong Agricultural University Tai’an, Shandong, 271018 China; 3 College of Forestry Engineering Shandong Agriculture and Engineering University, Ji’nan, Shandong, 250000 China

**Keywords:** Apple replant disease, Apple rootstock, Rhizosphere microorganism, *Bacillus*, *Fusarium*

## Abstract

**Background:**

The rhizosphere microbiome, as the second genome of plant immunity, forms a critical ecological barrier in plant-pathogen interactions. However, its functional mechanism in resisting the replanting disease pathogenic *Fusarium proliferatum* MR5 in apples has not been systematically elucidated. This study employed an integrated multi-omics approach to investigate the rhizosphere mechanisms of resistant (CG935) and sensitive (M9T337) apple rootstocks, aiming to uncover the metabolic and microbial interactions underlying apple replant disease resistance.

**Results:**

Multiple omics joint analysis found that the infection of *Fusarium proliferatum* MR5 triggered the activation of a specific lysine biosynthesis pathway in resistant rootstocks, and the expression levels of key rate limiting enzymes aspartate kinase and dihydrodipicolinate synthase were significantly upregulated by 2.79 ~ 6.81 times compared to M9T337. Along with the metabolic reprogramming process, the efflux of lysine from the rhizosphere increased, and *Bacillus* with broad-spectrum antibacterial activity were specifically recruited, increasing its relative abundance by 40.73%. In vitro assays demonstrated that the recruited *Bacillus* suppressed *Fusarium* spore germination and disrupted mycelial growth through the production of antifungal compounds, including 2,4-di-tert-butylphenol and bacillomycin. Potted experiments have confirmed that the synergistic treatment of *Bacillus* and lysine significantly reduces the number of pathogenic *Fusarium* in the rhizosphere, increases soil enzyme activity, and reshapes a more stable rhizosphere bacterial community structure by enhancing the modularity (the degree of modularity in microbial network structure) of the microbial network. This collaborative strategy effectively alleviates the physiological damage of apple seedlings under replanting stress, resulting in a 31.18% increase in plant fresh weight. Field validation experiments further demonstrate that this strategy can promote the growth of replanted apple saplings and reduce the occurrence of apple replant disease.

**Conclusions:**

Our findings elucidate an apple replant disease resistance mechanism in apple rootstocks involving lysine-mediated recruitment of protective *Bacillus*, which enhances rhizosphere microbiome stability and suppresses soil pathogenic *Fusarium*. Developed a technology for synergistic control of apple replant disease using *Bacillus*-lysine. The research results provide theoretical basis and practical solutions for green control of apple replant disease based on precise regulation of rhizosphere microbiome.

Video Abstract

**Supplementary Information:**

The online version contains supplementary material available at 10.1186/s40168-025-02301-9.

## Introduction

Apples are a widely cultivated fruit tree, and they have been grown in Central Asia since 1500 BC [1]. Approximately 89 million tons of apples are produced annually, and it has become the world’s third most popular fruit. Apple production has greatly contributed to the economic development of various regions (http://www.fao.org/faostat/en/#data/QC). The value of apple trees declines over their life; consequently, old apple trees are often replaced with new trees. However, planting apple saplings on land where apples were previously grown can lead to the development of apple replant disease (ARD). These main signs of ARD include the stagnation of root growth, root necrosis, plant dwarfing, increases in disease and insect pests, low fruit yield, and decreased fruit quality [2, 3]. ARD has been reported to occur in most apple-growing regions of the world. ARD in the USA reduces the yield per hectare in the first 3 years of orchard production by $2000 to $70,000 [4, 5]. In South Africa, the loss of orchards due to ARD can be as high as 50% over the entire life cycle [6]. The problem will worsen as suitable land that was not previously used for apple cultivation becomes increasingly scarce. In China alone, more than 1 million hectares of apple orchards are estimated to be affected by ARD. Previous studies have shown that ARD mainly occured because special pathogens affect rhizosphere microorganisms and cause root infection [7, 8]. In our previous study, *Fusarium proliferatum*, *F. moniliforme*, *F. oxysporum*, *F. solani*, and *F. proliferatum* f. sp *malus domestica* (*Fpmd* MR5) were identified as pathogens that caused ARD in China [9, 10]. There is thus a need to clarify how ARD can be controlled via studying of rhizosphere microorganisms.

The rhizosphere of plants is the most active interface for the exchange of materials between roots and soil, and a large number of highly complex microorganisms, namely rhizosphere microorganisms, inhabit this interface [11, 12]. The rhizosphere microbiome has been referred as the second genome of plants [13, 14]. The unique rhizosphere microorganism communities of plants enhance their resistance to stress [15, 16]. For example, specific bacterial species in the rhizosphere are enriched in disease-resistant beans to inhibit pathogens [17]. Beneficial microorganisms in plants are selectively recruited following exposure to pathogens. When *Arabidopsis thaliana* leaves are infected with *Hyaloperonospora arabidopsidis*, the downy mildew pathogen, the roots recruit *Microbacterium*, *Stenotrophomonas*, and *Xanthomonas* spp. to improve their resistance [18]. Most studies of the rhizosphere microorganisms that contribute to enhancing the resistance of plants to pathogen infection have mainly focused on tomato [19], banana [20], *Astragalus mongholicus* [21], peanut [22], corn [23], and other herbs. Studies of whether rhizosphere microorganisms enhance resistance to pathogen infection in apple are lacking, yet such studies are needed to facilitate future efforts to manipulate rhizosphere microorganisms and mitigate ARD.

Plant rhizosphere microorganism community assembly is a non-random process that is largely influenced by rhizosphere metabolites [24]. Rhizosphere metabolites mainly include sugars, amino acids, and fatty acids [25, 26]. When plants are subjected to stress, they recruit specific microorganisms to maintain their healthy growth by altering the composition of rhizosphere metabolites [27, 28]. For example, tomatoes secrete two key metabolites (riboflavin and 3-hydroxyflavone) that increase resistance to *Ralstonia solanacearum* by recruiting *Streptomyces* [29]. Cucumber roots secrete citric acid, pyruvate acid, succinic acid, and fumarate. The recruitment of *Comamonadaceae* inhibits the growth of *F. oxysporum* [30]. Wild soybeans are resistant to salt stress by secreting key metabolites, namely purines, to recruit beneficial *Pseudomonas* bacteria [31]. Previous researchers have referred to this response of plants under stress as a “cry for help” [32]. However, it remains unclear whether signaling substances such as rhizosphere metabolites are released in various resistant plants, including woody plants, to recruit functional microorganisms that inhibit pathogens. Clarifying the role of rhizosphere microorganisms in enhancing pathogen resistance will be critically important for the future development of measures to control ARD.

Here, we hypothesized that the roots of apple recruit specific microorganisms to enhance their resistance to pathogens under pathogen infection, and this recruitment is mediated by key rhizosphere metabolites. To test this hypothesis, we used 16S rRNA gene amplification sequencing to study changes in the composition and function of root bacterial communities of resistant and sensitive apple rootstocks under *Fpmd* MR5 stress, isolated and identified specific microorganisms recruited by resistant apple rootstocks, and studied their inhibition mechanism against pathogenic *Fusarium*. Subsequently, we used non-targeted metabolomics to identify key rhizosphere metabolites that recruit specific microbes. Finally, we conducted pot and field experiments to determine the effect of the combined application of specific root-recruited microorganisms and key metabolites for the control of ARD. Our study provides insights into how the release of metabolites by plants recruits specific microorganisms that increase their pathogen resistance; our findings will also aid the future development of ARD control measures.

## Results

### *Fpmd* MR5 inhibits the growth of both rootstocks

**Fig. 1:**
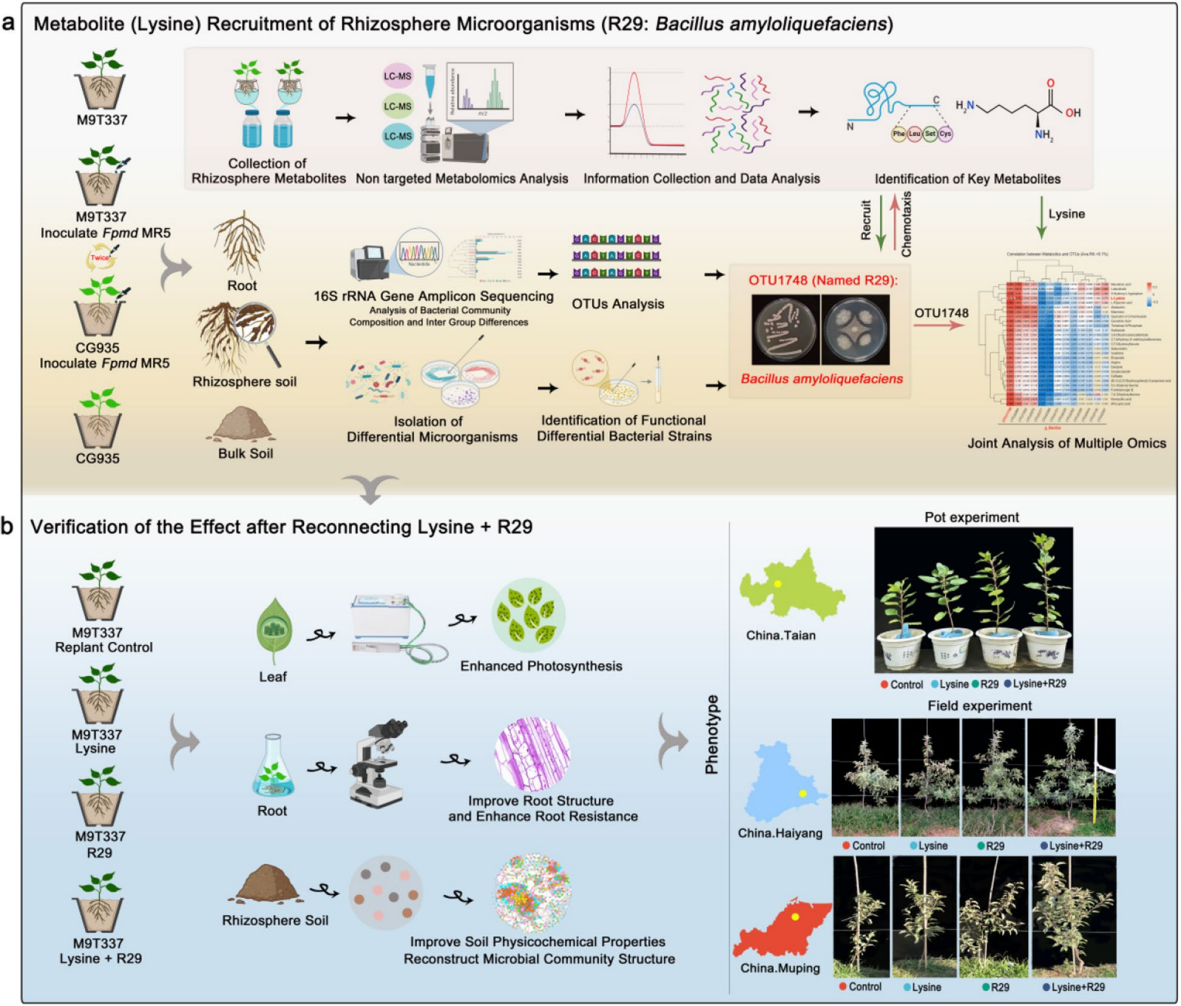
Mechanism underlying the specific recruitment of apple rhizosphere microorganisms under *Fpmd* MR5 stress and experimental flow chart of pot and field experiments. **a** Specific microorganisms recruited by the rhizosphere of CG935 and M9T337 under *Fpmd* MR5 stress and identification of key rhizosphere metabolites for the recruitment of specific microbes. **b** The effect of specific microorganisms and key rhizosphere metabolites recruited to control ARD under pot and field conditions. CG935, resistant rootstock; M9T337, sensitive rootstock

**Fig. 2:**
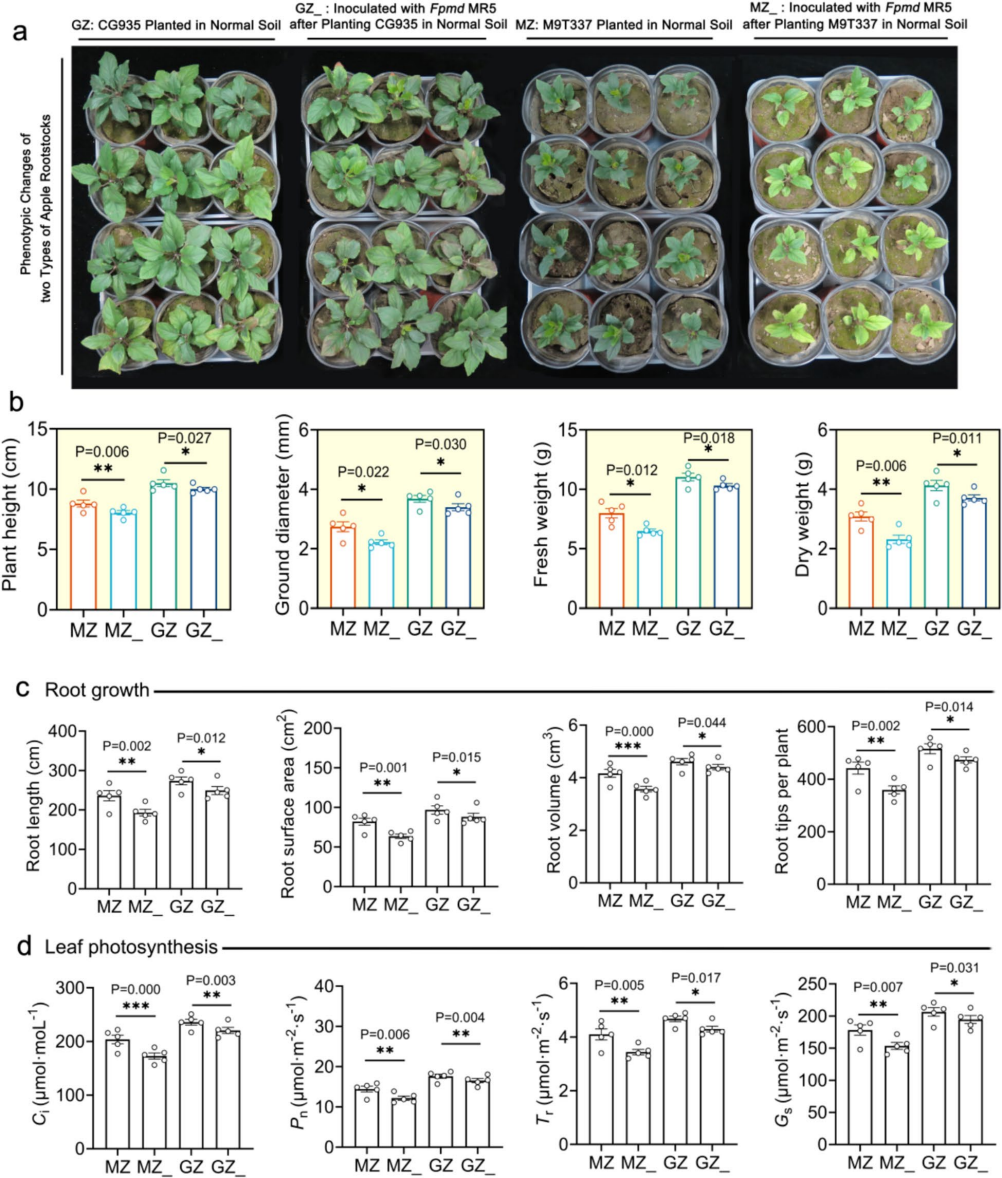
Effects of *Fpmd* MR5 on the growth of CG935 and M9T337. **a** Phenotypes of CG935 and M9T337 under *Fpmd* MR5 stress. **b** Plant height, ground diameter, fresh weight, and dry weight of CG935 and M9T337 under *Fpmd* MR5 stress. **c** Root length, root surface area, root volume, and number of root tips of CG935 and M9T337 under *Fpmd* MR5 stress. **d** Intercellular CO_2_ concentration (*C*_i_), stomatal conductivity (*G*_s_), net photosynthetic rate (*P*_n_), and transpiration rate (*T*_r_) of CG935 and M9T337 under *Fpmd* MR5 stress. The asterisk on the bars indicates significant differences between the two groups as measured via a two-tailed Student’s *t*-test (*0.01 < *P* ≤ 0.05, **0.001 < *P* ≤ 0.01, ***P ≤ 0.001) and the mean ± SEM (*n* = 5) for each histogram. CG935, resistant rootstock; M9T337, sensitive rootstock. MZ, M9T337 planted in normal soil; MZ_, inoculated with *Fpmd* MR5 after planting M9T337 in normal soil; GZ, CG935 planted in normal soil; GZ_, inoculated with *Fpmd* MR5 after planting CG935 in normal soil

In the USA, M9T337 has been identified as a sensitive rootstock and CG935 has been identified as a resistant rootstock [33], but whether the two rootstocks are resistant and sensitive to *Fpmd* MR5 is unclear. In order to assess the response of CG935 and M9T337 to *Fpmd* MR5, to better demonstrate that CG935 is *Fpmd* MR5-resistant rootstock and M9T337 is *Fpmd* MR5-sensitive rootstock, pot experiments were conducted to determine plant biomass, leaf photosynthetic parameters, root structure, and root protective enzyme activity, which can affect the plant growth status (Fig. 1a). *Fpmd* MR5 inoculation significantly reduced both biomass and photosynthetic parameters in both rootstocks, with a consistently stronger inhibitory effect on M9T337 than on CG935 (Fig. 2a, b, d). Similarly, root growth and protective enzyme activities were more severely suppressed in M9T337 (Fig. 2c and Supplementary Fig. 1). To investigate the potential role of soil microorganisms in this resistance, we repeated the experiment in sterilized soil. Soil sterilization substantially enhanced the inhibitory effect of *Fpmd* MR5 on biomass in both rootstocks (Supplementary Fig. 2). Collectively, these results confirm that M9T337 is sensitive to *Fpmd* MR5, whereas CG935 is resistant, and that this resistance is likely mediated by soil microorganisms.

### *Fpmd* MR5 altered root-associated bacterial community structure and function in both rootstocks

**Fig. 3:**
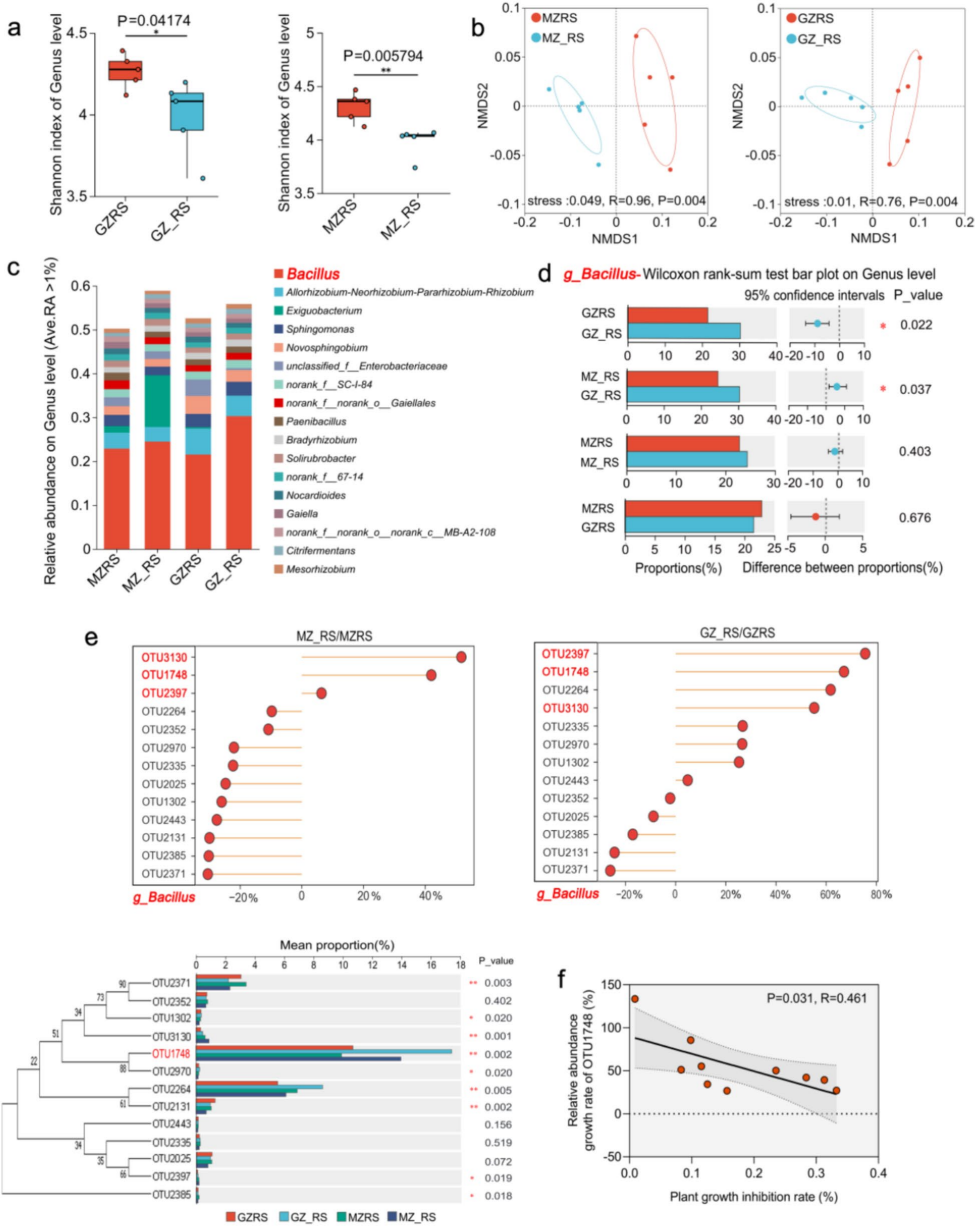
Changes in the rhizosphere bacterial community structure of CG935 and M9T337 under *Fpmd* MR5 stress.** a** Rhizosphere bacterial community Shannon index of CG935 and M9T337 under *Fpmd* MR5 stress. **b** β diversity of rhizosphere bacterial communities in CG935 and M9T337 under *Fpmd* MR5 stress. **c** Rhizosphere bacterial community composition of CG935 and M9T337 under *Fpmd* MR5 stress (genus level). **d** Analysis of the effects of *Fpmd* MR5 stress on differences in the RA of *Bacillus* in the rhizosphere soil of CG935 and M9T337. **e** Analysis of differences in the relative abundance of OTUs (relative abundance > 0.1%) in rhizosphere *Bacillus* CG935 and M9T337 under *Fpmd* MR5 stress.** f** Correlation analysis of the relative abundance of OTU1748 and the plant growth inhibition rate. The asterisks on the bars indicate significant differences between the two groups according to Student’s *t*-test (*0.01 < *P* ≤ 0.05, **0.001 < *P* ≤ 0.01, ***P ≤ 0.001) and the mean ± SEM (*n* = 5) for each histogram. CG935, resistant rootstock; M9T337, sensitive rootstock; MZRS, MZ-treated rhizosphere soil; MZ_RS, MZ_-treated rhizosphere soil; GZRS, GZ-treated rhizosphere soil; GZ_RS, GZ_-treated rhizosphere soil

To characterize the effect of *Fpmd* MR5 on CG935 and M9T337 root-related bacteria, we conducted 16S rRNA gene amplification sequencing and obtained 2,721,221 high-quality sequences. The bacterial Shannon index was highest in bulk soil, intermediate in the rhizosphere soil, and lowest in roots, a pattern maintained after *Fpmd* MR5 inoculation (Supplementary Fig. 3). However, inoculation significantly reduced Shannon index across all compartments, with the most pronounced decrease observed in the rhizosphere (MZRS&MZ_RS, *P* = 0.005794; GZRS&GZ_RS, *P* = 0.04174) (Fig. 3a and Supplementary Fig. 3). β diversity analysis showed that that *Fpmd* MR5 inoculation significantly altered the bacterial community structure, with the strongest effect again observed in the rhizosphere (MZRS&MZ_RS, *R* = 0.98, *P* = 0.004; GZRS&GZ_RS, *R* = 0.76, *P* = 0.004). Significant separation was observed among the rhizosphere bacterial communities (Fig. 3b), and bacterial communities in the bulk soil and roots were not well separated (Supplementary Fig. 4). These results suggest that *Fpmd* MR5 can induce the recruitment of specific microorganisms to the rhizosphere to alter bacterial community structure.

We next determined the types of microorganisms recruited by the rhizosphere of CG935 and M9T337 following induction by *Fpmd* MR5 by characterizing changes in microbial community structure. At the phylum level, Proteobacteria and Firmicutes dominated the rhizosphere. Inoculation with *Fpmd* MR5 consistently decreased the relative abundance (RA) of Proteobacteria and increased that of Firmicutes (Supplementary Fig. 5). We also found that the composition of bacterial genera in bulk soil, rhizosphere soil, and the roots differed among treatments (Fig. 3c and Supplementary Fig. 5). Analysis of rhizosphere-enriched genera revealed a higher number in the CG935 (120 genera) than in the M9T337 (108 genera). Subsequently, we found that 50 genera were co-enriched (referring to the synchronous and significant increase in abundance of two or more microbial taxa under the same treatment conditions) in CG935 and M9T337 rhizosphere. Among 50 genera co-enriched in both rootstocks, 15 showed significant relative abundance changes upon inoculation. Notably, *Bacillus* exhibited the greatest increase in relative abundance in the CG935 rhizosphere (GZ_RS) and became the most dominant genus (Fig. 3d). This increase was significant in CG935 (GZ_RS VS GZRS, *P* = 0.022) but not in M9T337 (MZ_RS vs MZRS, *P* = 0.403). Next, the relative abundance of OTUs (relative abundance > 0.1%) in *Bacillus* was analyzed. After inoculation with *Fpmd* MR5, OTU3130, OTU1748, and OTU2397 in the rhizosphere of CG935 and M9T337 were significantly enriched (Fig. 3e). We analyzed correlations of the relative abundance of OTU3130, OTU1748, and OTU2397 with the dry weight inhibition rate of plants (Fig. 3f and Supplementary Fig. 6) and found a significant negative correlation between OTU1748 and the dry weight inhibition rate (*R* = 0.456, *P* = 0.032). There was no significant correlation between OTU3130 and OTU2397 (OTU3130, *R* = 0.001, *P* = 0.931; OTU2397, *R* = 0.0.268, *P* = 0.125). Finally, co-occurrence network analysis indicated that *Fpmd* MR5 inoculation enhanced the complexity and stability of the rhizosphere bacterial network in the CG935. The CG935 network possessed a higher ratio of positive-to-negative correlations, along with increases in the number of edges (26.87%), average degree (22.35%), and average clustering coefficient (2.86%) compared to the M9T337 network. *Bacillus* was a central hub in this stabilized network, which was dominated by Firmicutes and Proteobacteria (Supplementary Fig. 7). Therefore, we speculate that the stability of community structure was enhanced in resistant rootstocks by recruiting more *Bacillus* OTU1748, which reduced the stress of *Fpmd* MR5.

### *Fpmd* MR5 altered the functions and metabolite composition of rhizosphere bacterial communities in both rootstocks

**Fig. 4:**
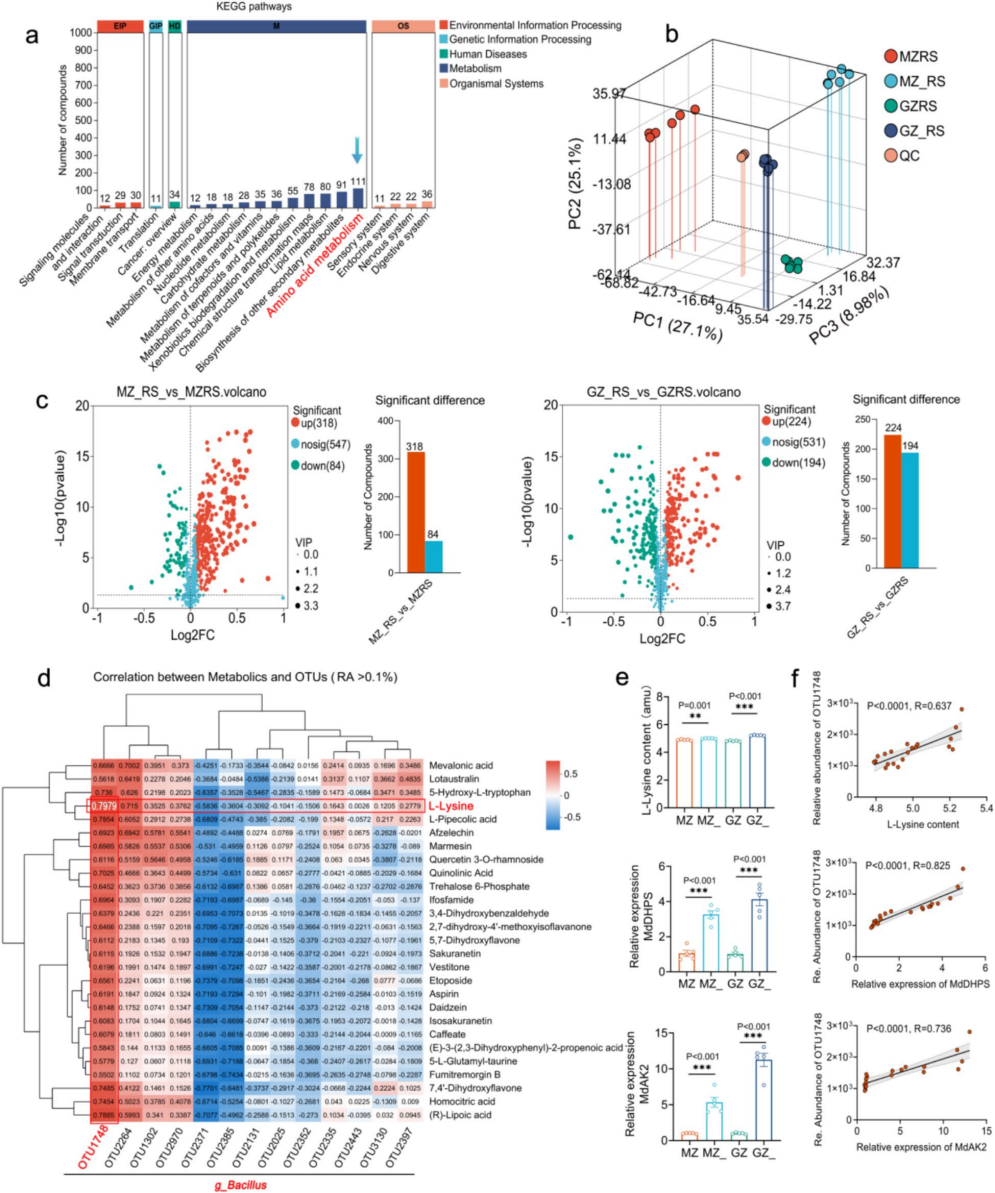
Changes in the functions and rhizosphere metabolites of the rhizosphere bacterial community of CG935 and M9T337 under *Fpmd* MR5 stress.** a** Rhizosphere bacterial community functions of CG935 and M9T337 under *Fpmd* MR5 stress.** b** Principal component analysis of the rhizosphere metabolites of CG935 and M9T337 under *Fpmd* MR5 stress. **c** Significant differences in the rhizosphere metabolites of CG935 and M9T337 under *Fpmd* MR5 stress. **d** Heat maps showing increases in rhizosphere metabolites and OTUs of CG935 and M9T337 under *Fpmd* MR5 stress. **e** Rhizosphere lysine content and relative expression of MdDHPS and MdAK2 under *Fpmd* MR5 stress. **f** Correlations of the rhizosphere lysine content, relative expression of MdDHPS and MdAK2, and relative abundance of OTU1748. The asterisks on the bars indicate significant differences between the two groups according to a two-tailed Student’s *t*-test (*0.01 < *P* ≤ 0.05, **0.001 < *P* ≤ 0.01, ***P ≤ 0.001) and the mean ± SEM (*n* = 5) for each histogram. CG935, resistant rootstock; M9T337, sensitive rootstock; MZ, M9T337 planted in normal soil; MZ_, inoculated with *Fpmd* MR5 after planting M9T337 in normal soil; GZ, CG935 planted in normal soil; GZ_, inoculated with *Fpmd* MR5 after planting CG935 in normal soil; MZRS, MZ-treated rhizosphere soil; MZ_RS, MZ_-treated rhizosphere soil; GZRS, GZ-treated rhizosphere soil; GZ_RS, GZ_-treated rhizosphere soil

Rhizosphere metabolites are involved in the enrichment of rhizosphere microorganisms [31]. To identify the key rhizosphere metabolites involved in the enrichment of rhizosphere *Bacillus*, we investigated the function of rhizosphere bacterial communities. *Fpmd* MR5 can alter the rhizosphere bacterial community function of CG935 and M9T337 to different degrees. The relative abundance of metabolic functions in the KEGG1 pathways was the highest. Next, we analyzed metabolic functions and found that the relative abundance of amino acid metabolism in the KEGG2 pathways was the highest. Finally, we analyzed the amino acid metabolism function in the KEGG3 pathways and detected tryptophan metabolism, lysine degradation, phenylalanine metabolism, and other functions (Fig. 4a and Supplementary Fig. 8 and Table 1).

Next, we used non-targeted metabolomics to determine the rhizosphere metabolites of CG935 and M9T337. The types and amounts of rhizosphere metabolites differed in CG935 and M9T337 inoculated and uninoculated with *Fpmd* MR5 (Fig. 4b). Upon *Fpmd* MR5 inoculation, the number of detected metabolites increased in both rootstocks, with CG935 consistently exhibiting a greater metabolic diversity than M9T337 (Supplementary Fig. 9). Differential abundance analysis identified numerous significantly altered metabolites: in CG935, 224 were upregulated and 194 were downregulated; in M9T337, 318 were upregulated and 84 were downregulated (Fig. 4c). According to previous studies, increases in the relative abundance of rhizosphere microorganisms can be inferred based on increases in the content of rhizosphere metabolites [31]. We analyzed the relative abundance of rhizosphere microorganisms in two comparison groups: GZ_RS VS GZRS and GZ_RS VS MZ_RS (the sign “_” for the treatments inoculated with *Fpmd* MR5). A total of 27 rhizosphere metabolites were co-enriched in both groups (Supplementary Table 2). Correlations of 27 rhizosphere metabolites with rhizosphere bacteria (genus level) and OTUs (relative abundance > 0.1%) within *Bacillus* were analyzed. We detected positive correlations between *Bacillus*, OTU1748, and 27 rhizosphere metabolites, and OTU1748 was most strongly positively correlated with lysine (Fig. 4d and Supplementary Fig. 10). Previous microbiome studies have shown that the relative abundance of amino acid metabolism was highest in the rhizosphere bacterial community of CG935; among the 27 different rhizosphere metabolites detected by non-targeted metabolomics, 7 different rhizosphere metabolites were detected in the amino acid metabolism pathway in the microbiome (Supplementary Table 3). Combined analysis of the amino acid metabolism function and 7 different rhizosphere metabolites of rhizosphere bacterial communities revealed that lysine may be a key rhizosphere metabolite for recruiting *Bacillus* to the rhizosphere.

To identify the cause of the increase in lysine in the rhizosphere of CG935 and M9T337, we conducted a quantitative analysis of the genes encoding dihydrodipicolinate synthase (DHDPS) and aspartate kinase (AK) involved in lysine synthesis. The results showed that the expression levels of these genes in CG935 and M9T337 were significantly increased after inoculation with *Fpmd* MR5, and their expression levels were significantly higher in CG935 than in M9T337 (Fig. 4e). The lysine content, MdDHDPS expression, and MdAK expression were significantly positively correlated with the relative abundance of OTU1748 (Fig. 4f; lysine, *R* = 0.637, *P* < 0.0001; MdDHDPS, *R* = 0.825, *P* < 0.0001; MdAK, *R* = 0.736, *P* < 0.0001). These results indicate that more lysine was may secreted by apple rootstocks under *Fpmd* MR5 stress by enhancing lysine biosynthesis; the accumulation of lysine then resulted in the recruitment of *Bacillus*.

### Isolation, identification, and functional evaluation of *Bacillus*

**Fig. 5:**
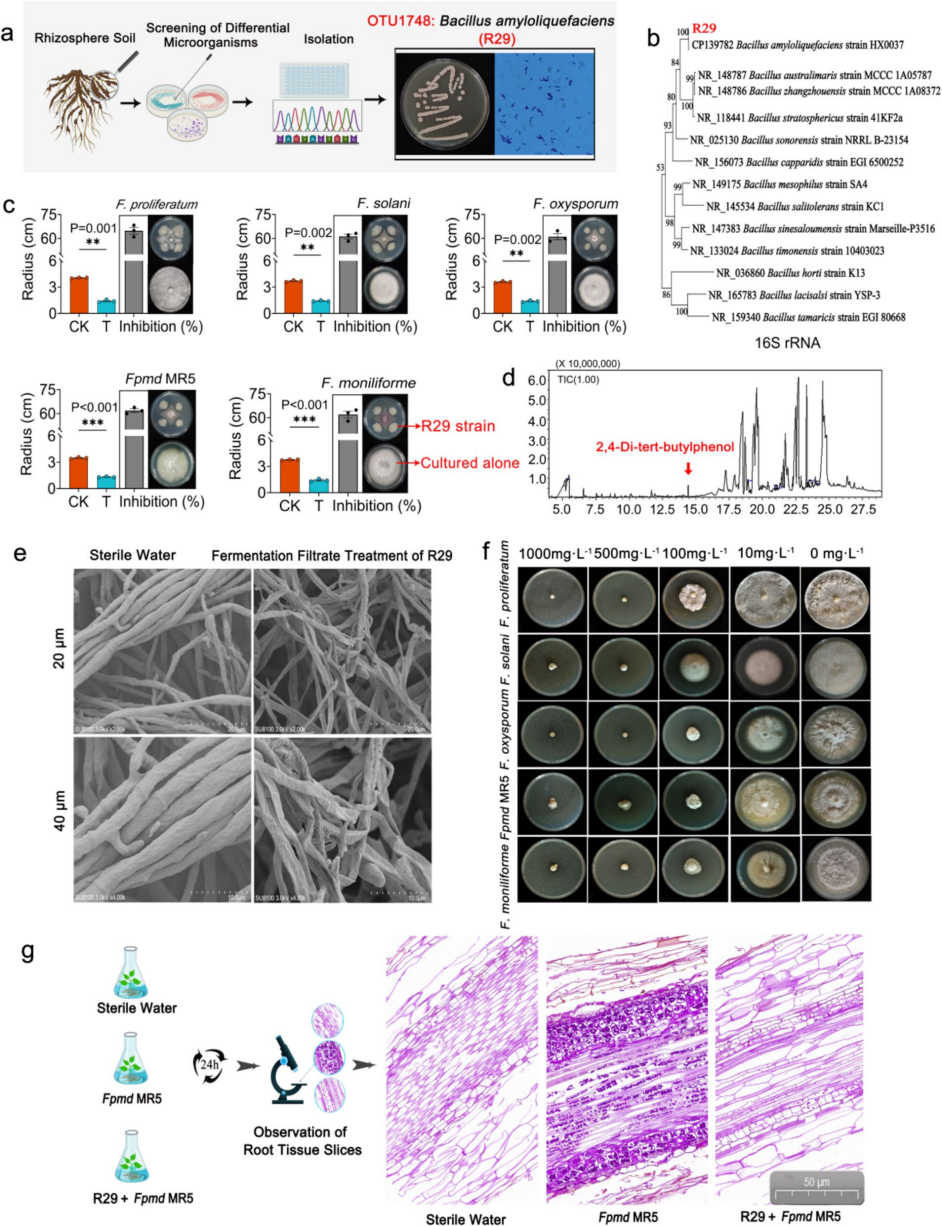
Isolation, identification, and functional evaluation of rhizosphere *Bacillus*.** a** Isolation of strain R29. **b** Identification of the 16S rRNA gene sequence of strain R29. **c** Effects of strain R29 on the growth of *F. proliferatum*, *F. solani*, *F. oxysporum*, *Fpmd* MR5, and *F. moniliforme*. **d** Identification of volatile substances of strain R29. **e** Effect of strain R29 on the mycelial morphology of *Fpmd* MR5. **f** Effects of 2, 4-di-tert-butylphenol on the growth of *F. proliferatum*, *F. solani*, *F. oxysporum*, *Fpmd* MR5, and *F. moniliforme*. **g** Protective effect of strain R29 on plant roots. The asterisk on the bars indicates significant differences between the two groups as measured by a two-tailed Student’s *t*-test (*0.01 < *P* ≤ 0.05, **0.001 < *P* ≤ 0.01, ***P ≤ 0.001) and the mean ± SEM (*n* = 5) for each histogram

To determine whether the highly enriched OTU1748 can enhance the resistance of plants to *Fpmd* MR5 stress, rhizosphere bacteria were isolated, and a strain of *Bacillus* R29 with 96.57% similarity to OTU1748 was obtained (Fig. 5a and Supplementary Fig. 11). Strain R29, a Gram-positive bacterium forming opaque, milky, and wrinkled colonies, exhibited protease and amylase activity (Fig. 5a, Supplementary Table 4). Based on morphological, physiological, biochemical, and phylogenetic analyses of the 16S rRNA and *gyr*A genes, strain R29 was identified as *Bacillus amyloliquefaciens* (Fig. 5b, Supplementary Fig. 12).

The inhibition rates of strain R29 against *F. proliferatum*, *F. moniliforme*, *F. oxysporum*, *F. solani*, and *Fpmd* MR5 were 64.76%, 61.97%, 61.07%, 61.22%, and 61.88%, respectively (Fig. 5c). The fermentation filtrate of strain R29 could not only inhibit the germination of pathogenic *Fusarium* spores but also significantly inhibit the growth of pathogenic *Fusarium* (Supplementary Fig. 13 and Supplementary Table 5). Scanning electron microscopy revealed that the filtrate caused severe structural damage to *Fpmd* MR5 mycelia, including breakage, bending, folding, and pore formation, leading to cell content leakage, whereas mycelia treated with sterile water remained intact (Fig. 5e).

To determine the volatile and non-volatile substances produced by strain R29 to inhibit *Fusarium*, we first analyzed the antibiotic synthesis genes of strain R29. Strain R29 could produce bacillomycin, iturins, subtilisin, fengycins, and surfactins (Supplementary Fig. 14). Subsequently, we used GC–MS to determine the volatile substances produced by strain R29 (Fig. 5d) and analyzed the substances in the 60 most abundant peaks. The results showed that the substances identified were mainly alkanes, alcohols, phenols, and esters (Supplementary Table 6). Given that some of these substances are harmful to humans, we selected six substances that are harmless to humans that could be identified for bacteriostatic tests. The results showed that 2, 4-di-tert-butylphenol strongly inhibited the pathogen *Fusarium*. When the concentration was 100 mg·L^−1^, the inhibition rates of *F. proliferatum*, *F. solani*, *F. oxysporum*, *Fpmd* MR5, and *F. moniliforme* were 62.59%, 49.57%, 85.20%, 80.14%, and 80.36%, respectively (Fig. 5f). The antibacterial effect of five of the other substances varied (Supplementary Fig. 15).

To confirm that strain R29 can protect apple roots and reduce the infection of *Fpmd* MR5, we generated slices of the apple root system for observation. The results showed that the roots were complete and neat under aseptic water treatment. The root epidermal cells inoculated with *Fpmd* MR5 were deformed, broken, shed, and arranged irregularly, and the mycelium invaded the cortical cells and entered the vascular column. New mycelia were mainly observed near the cell wall, and mature mycelia were scattered within the cell. Cortical cells and vascular columns were full of sticky substances and starch particles. The root epidermal and cortical cells inoculated with *Fpmd* MR5 after fermentation solution pretreatment of strain R29 were slightly contracted and ruptured; the mycelium was only attached to the root epidermal cells; and the cortical cells and vascular columns had low amounts of sticky substances and starch particles (Fig. 5g). These results indicate that the acquisition of resistance in rootstocks may depend on the ability of plants to recruit *Bacillus* to the rhizosphere.

### Interaction between lysine and strain R29 and its contribution to controlling ARD

**Fig. 6:**
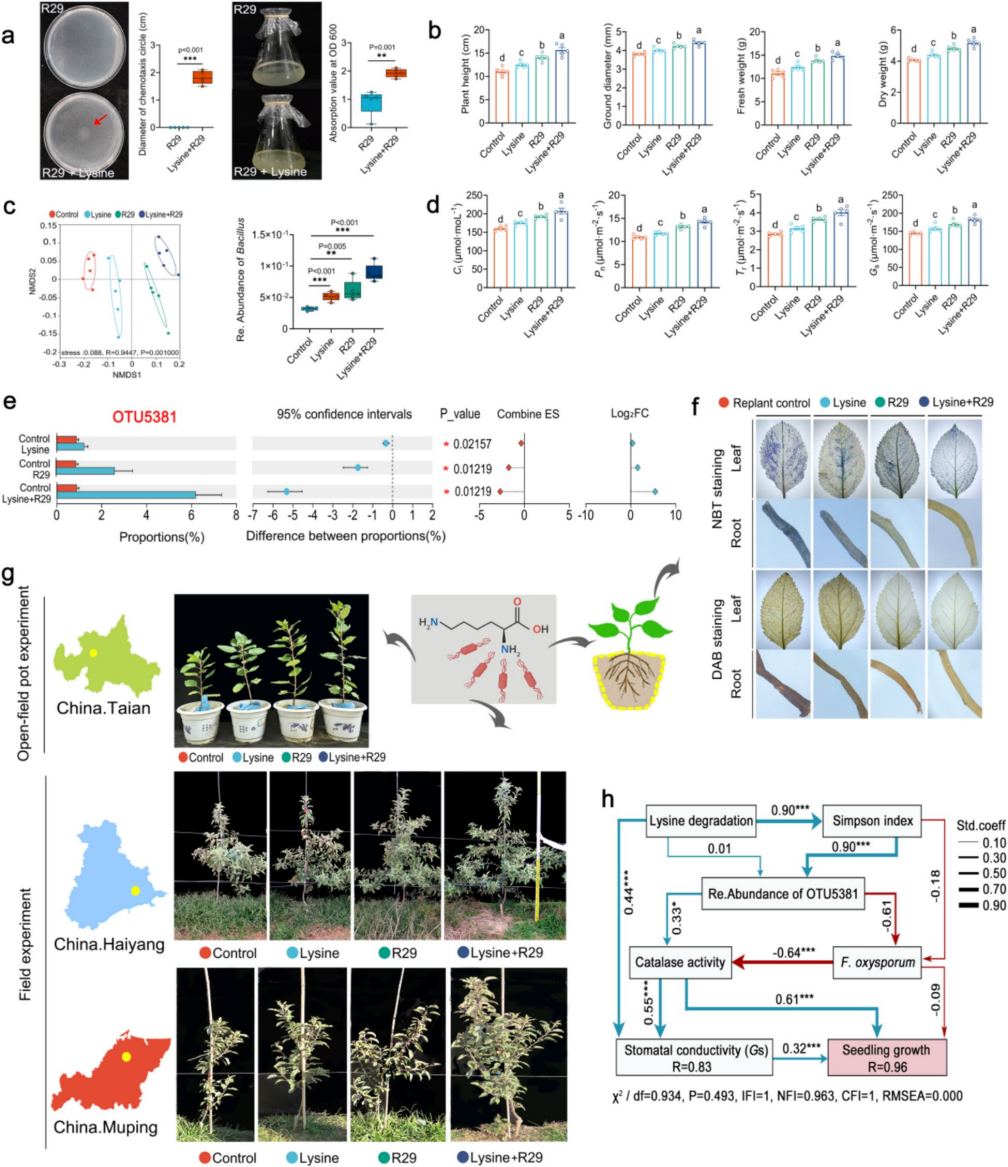
Evaluation of the control effect of strain R29 combined with lysine on ARD.** a** Utilization and chemotactic effect of lysine by strain R29. **b** Effects of lysine and strain R29 on the plant height, ground diameter, fresh weight, and dry weight of replanted M9T337. **c** Effects of lysine and strain R29 on the β diversity of rhizosphere bacterial communities. **d** The effects of lysine and strain R29 on the *C*_i_, *G*_s_, *P*_n_, and *T*_r_ of replanted M9T337. **e** Effects of lysine and strain R29 on the RA of rhizosphere *Bacillus*. **f** DAB and NBT staining of M9T337 leaves and roots. **g** Phenotypes of open-field pot and field (2024) plants treated with lysine and strain R29. **h** Effects of the lysine degradation function, Simpson index, relative abundance of OTU5381, soil enzyme activity, number of *Fusarium*, and *G*_s_ on plant growth. Blue arrows indicate positive effects; red arrows indicate negative effects. The asterisk on the bars indicates significant differences between the two groups according to two-tailed Student’s *t*-test (*0.01 < *P* ≤ 0.05, **0.001 < *P* ≤ 0.01, ***P ≤ 0.001), and different letters on the bars indicate significant differences between treatments (*P* < 0.05) by one-way ANOVA. Mean ± SEM for each histogram (*n* = 5)

Based on the results of 16S rRNA gene amplification sequencing and non-targeted metabolome analysis, we speculated that the roots of apple rootstock under *Fpmd* MR5 stress secrete lysine and then recruit *Bacillus* to protect the root system. To test this hypothesis, we co-cultured strain R29 with lysine. The addition of lysine significantly increased the OD600 of strain R29 (Fig. 6a), indicating that lysine could be utilized by strain R29. At the same time, a clear chemotactic ring was formed after adding lysine to the medium, and no chemotactic ring was formed in the blank control (Fig. 6a), indicating that lysine could be used as a signal substance to recruit strains.

We then evaluated the efficacy of lysine and R29 in controlling ARD through greenhouse, open field pot, and field experiments (Fig. 1b). First, we conducted a preliminary test to determine the optimal added concentration of lysine. Five concentrations of 0, 0.1‰, 0.2‰, 0.5‰, and 1‰ were tested. A concentration of 0.5‰ could improve plant growth to the greatest extent (Supplementary Fig. 16); thus, a lysine concentration of 0.5‰ was used in the subsequent experiment. Next, we found that lysine and strain R29 could promote plant growth to different degrees in the greenhouse pot experiment (Fig. 6b, g, and Supplementary Figs. 17, 26). The effect was strongest in the lysine + strain R29 treatment, followed by the strain R29 treatment and lysine treatment. This effect was replicated in open field pot and field experiments (Fig. 6g). We then determined the photosynthetic parameters (*C*_i_, *G*_s_, *P*_n_, and *T*_r_) in the leaves of replanted plants. Treatment with lysine + strain R29 enhanced these parameters (Fig. 6d).

We also performed DAB and NBT staining on the leaves and roots of the plants; DAB staining showed that the leaves and roots of the replanted control showed a large area of yellowish-brown precipitation, leaf-edge necrosis, and root tip wilting. In the lysine treatment, a small amount of yellowish-brown precipitation was observed, but it did not cover a large area. An obvious yellowish-brown precipitate was not observed in the strain R29 treatment and the lysine + strain R29 treatment. The results of NBT staining were similar to those of DAB staining. Large areas of blue spots were present in the leaves and roots of the replant control, and a small amount of blue spots were present in the R29 treatment and the lysine + strain R29 treatment (Fig. 6f). Subsequently, superoxide dismutase (SOD), peroxidase (POD), and catalase (CAT) activities and the malondialdehyde (MDA) content were measured. The lysine + strain R29 treatment could significantly increase the activities of SOD, POD, and CAT in the root system and reduce the MDA content in the root system (Supplementary Fig. 18).

In light of the previous analysis of the plant growth status, we initially determined that the combined use of lysine and strain R29 could significantly reduce the inhibitory effect of replanting on plants. To better determine the mechanism of the combined use of lysine and strain R29 to alleviate ARD, we analyzed the rhizosphere microecological environment. Analysis of the rhizosphere microbiome showed that the lysine + strain R29 treatment significantly increased the number of culturable bacteria and actinomycetes while reducing culturable fungi and the gene copy numbers of pathogenic *Fusarium* (Supplementary Figs. 19, 20). 16S rRNA gene sequencing revealed a distinct shift in the bacterial community structure following the combined treatment (Fig. 6c), which was associated with a significant increase in the relative abundance of *Bacillus* (Supplementary Figs. 21, 25). Notably, an OTU with > 93% similarity to R29 (OTU5381) became the most dominant *Bacillus* OTU in the lysine + strain R29 treatment (Fig. 6e and Supplementary Fig. 22). Co-occurrence network analysis indicated that this treatment fostered a more complex and stable bacterial community (Supplementary Fig. 23). Functional prediction revealed a significant enhancement of lysine degradation pathways, and soil enzyme activities were also highest in this treatment (Supplementary Fig. 24). The above findings were used to clarify the relationships between the relative abundance of OTU5381, the relative abundance of the lysine degradation function, Simpson index, soil enzyme activity, the number of *Fusarium*, *G*_s_, and plant growth using a structural equation model (Fig. 6h). The relative abundance of OTU5381, the relative abundance of the lysine degradation function, Simpson index, soil enzyme activity, and *G*_s_ were directly or indirectly positively correlated with plant growth, and the number of *Fusarium* was directly and negatively correlated with plant growth. In conclusion, these results suggest that lysine could regulate the rhizosphere colonization of strain R29 and reshape the rhizosphere microecological environment, thus alleviating the occurrence of ARD.

## Discussion

We investigated changes in root-associated microorganisms and rhizosphere metabolites induced by *Fpmd* MR5 in CG935 (*Fpmd* MR5-resistant apple rootstock) and M9T337 (*Fpmd* MR5-sensitive apple rootstock). Under *Fpmd* MR5 stress, the rhizosphere microorganisms of resistant rootstocks, including *Bacillus*, were highly enriched, and *Bacillus* inhibited the growth of pathogens by producing volatile and non-volatile substances. Finally, we demonstrated that the interaction of rhizosphere metabolites with enriched rhizosphere microorganisms confers resistance to rootstocks.

Rhizosphere microorganisms are the main factor underlying the plant defense response [34, 35]. After inoculation with *Fpmd* MR5, bacterial diversity in bulk soil, rhizosphere, and the roots all decreased to varying degrees; among them, the bacterial diversity in the rhizosphere decreased the most significantly. This is consistent with the microbial response patterns of crops such as Astragalus and soybeans after being infected by *Fusarium* [21, 36, 37], indicating that the competition and disruption of pathogens on the rhizosphere micro-ecosystem are universal. However, compared with the sensitive rootstocks, the resistant rootstocks formed a microbial network under stress that was centered on the Firmicutes and Proteobacteria phyla, with a significantly enhanced positive correlation between species. This finding is consistent with ecological theory, which states that microbial communities with stronger positive interactions typically have higher stability and resistance to disturbances [38]. Similar phenomena have also been observed in the rhizosphere microbial networks of wheat disease-resistant varieties. Furthermore, the relative abundance of *Bacillus* in the rhizosphere of resistant rootstocks was significantly higher than that of sensitive rootstocks, indicating that they have the ability to actively recruit beneficial microorganisms. This strategy has been reported in the root-associated immunity of various plants [39].

Isolation of rhizosphere bacteria and tests of their antifungal ability showed that *Bacillus* R29 could effectively antagonize the pathogen *Fusarium*. The fermentation solution of strain R29 could inhibit the growth and reproduction of pathogen *Fusarium* by reducing the growth rate of mycelia, destroying the integrity of mycelia, and hindering spore germination, which is consistent with the mechanism known for *Bacillus* to inhibit fungal growth by producing lipopeptide antibiotics [40]. Genomic analysis confirmed that R29 contains synthetic genes such as bacillomycin, iturins, and fengycins, and these compounds have been widely reported to induce fungal cell apoptosis [40]. We also found that strain R29 can significantly inhibit the growth of pathogenic *Fusarium* when not directly exposed to it. Previous studies have shown that biocontrol strains can employ more than one antimicrobial mechanism [41]. *Bacillus* can produce volatile organic compounds to inhibit the growth and spore germination of fungal pathogens [42]. We also found that 2, 4-di-tert-butylphenol produced by strain R29 could significantly inhibit the growth of *Fusarium*, and some of the substances had no antibacterial effect, which may be attributed to the synergistic effect of these substances as synthetic precursors of antibacterial compounds. This compound may also induce resistance in plants [43]. Therefore, as a strain with both volatile and non-volatile antibacterial capabilities, strain R29 has a multi-faceted biocontrol mechanism, which is in line with the current understanding of highly efficient biocontrol bacteria [39].

Plant rhizosphere metabolites comprise primary and secondary metabolites, and diversity in the chemical composition of plant rhizosphere metabolites alters the composition and function of microorganisms by affecting their activities [44, 45]. Metabolomics analysis revealed that under the stress of *Fpmd* MR5, the contents of 27 metabolites such as lysine in the rhizosphere of resistant rootstocks were significantly higher than those in sensitive rootstocks. Combined with the enrichment of amino acid metabolic pathways in the rhizosphere bacterial community, we hypothesized that lysine might be a key signaling molecule regulating the rhizosphere microenvironment of resistant rootstocks. This inference is consistent with findings in other crop systems: for instance, high-resistant banana varieties secrete metabolites to enrich beneficial fungi [46], while healthy root systems of watermelons recruit beneficial bacteria by secreting more palmitic acid [47]. Further studies have shown that after inoculation with *Fpmd* MR5, the expression of key enzymes involved in lysine synthesis in the root systems of resistant rootstocks was significantly upregulated, suggesting that they might actively secrete lysine into the rhizosphere [48]. In vitro experiments have confirmed that lysine can significantly promote the growth of strain R29. It has been reported that lysine can promote the growth of microorganisms and has become a necessary component in some microbial growth media [49]. In addition, lysine can cause strain R29 to form chemotaxis rings. This is similar to the mechanism in which organic acids in the root exudates of cucumbers and bananas attract beneficial *Bacillus* [50].

At present, many biocontrol strategies based on functional strains and metabolites have shown significant effects in the laboratory; their field application results are often unstable [14]. In our study, the combination of lysine and strain R29 could significantly promote the growth of plants based on potted and field experiments. This indicated that this treatment provided the optimal microecological environment for replanted soil [51]. Replanting causes an imbalance in soil microbial community structure [52]. After applying lysine and strain R29, the number of bacteria and actinomyces significantly increased, and the number of fungi and pathogen *Fusarium* significantly decreased in replanted soil. This might be because strain R29 can antagonize *Fusarium*, thus reducing its quantity [53]. On the other hand, lysine may enhance the colonization and reproduction of strain R29 in the rhizosphere, thereby increasing its competitiveness for nutrients and ecological niches, and thus indirectly inhibiting *Fusarium* [54]. The results of 16S rRNA gene amplicon sequencing indicated that strain R29 had the highest relative abundance in the mixed treatment, suggesting that strain R29 can colonize the apple rhizosphere [55]. Similarly, the combined application of lysine and strain R29 could increase the activities of soil enzymes to the greatest extent, which may explain why this treatment increased the number of beneficial microorganisms. Some studies have found that beneficial microorganisms in soil can improve soil enzyme activities [56]. We also found that the combination of lysine and strain R29 could increase the activities of SOD, POD, and CAT in roots and increase the photosynthetic parameters in leaves. Strain R29 might reduce the number of pathogenic *Fusarium* in soil and alleviate the stress of replanting on plants [57]. These effects might also be related to the use of lysine. Previous studies have shown that the catabolites of lysine indirectly produce tryptophan through the glycoside pathway [58], and tryptophan is involved in plant auxin synthesis [59]. Auxin is beneficial for promoting the formation of plant roots, enhancing plant growth, and improving resistance. Some studies have reported that the exogenous application of zinc lysine chelate can reduce the content of H_2_O_2_ and MDA in maize plants, and promote plant growth [60].

Through chemical kinetics and exogenous addition experiments in this study, the crucial role of lysine in mediating the *Bacillus* control of ARD was revealed, and it was preliminarily confirmed that it can reconstruct the beneficial microbial community. However, there are still several key knowledge gaps that need to be filled. Firstly, the dynamic process of the interaction between apple rootstock and *Fusarium* is not clear, and there is a lack of systematic temporal dimension analysis. Secondly, the molecular interaction mechanism between lysine and the rhizosphere microbial community, as well as how microbial activities precisely regulate the systemic resistance of the apple rootstock, have not been fully explained. Key links such as the recognition of lysine-specific microbial receptors and the signal network that precisely regulates the interaction among the microbial, rootstock, and *Fusarium* all need to be verified through subsequent experiments. Moreover, the field validation of the proposed control scheme in areas such as Haiyang and Muping in Shandong Province is still at a preliminary stage, and the long-term stability and functional persistence of the reconstituted microbial community still have uncertainties. Due to the lack of systematic data from multiple regions and seasons, it is difficult to assess the functional resilience and ecological adaptability of this microbial combination under different soil types and agricultural management practices.

To address these limitations, future research should be conducted in the following aspects: designing refined time series experiments, conducting systematic sampling at multiple consecutive time points after the interaction between rootstock and *Fusarium* occurs, integrating quantitative PCR, microbiome analysis, and non-targeted metabolomics techniques to dynamically track the relative abundance changes of *Bacillus* OTU1748, the expression patterns of lysine synthesis-related genes, and the temporal evolution of lysine concentration in the rhizosphere. At the same time, it is planned to collaborate with laboratories equipped with advanced technical platforms to directly verify the necessity of this metabolic pathway in its probiotic function by conducting experiments of inhibiting or overexpressing lysine biosynthesis at the plant level or by knocking out key functional genes in *Bacillus*. Additionally, longitudinal field trials spanning no less than 5 years across different production areas should be carried out to systematically evaluate the persistent colonization and functional maintenance capabilities of microbial communities under different ecological regions and agricultural management measures, thereby identifying the key environmental drivers affecting their functional stability.

## Conclusion

In summary, we used 16S rRNA gene amplification sequencing and non-targeted metabolomics to study changes in root-associated microorganisms and rhizosphere metabolites in different resistant apple rootstocks under *Fpmd* MR5 stress. Our results indicate that the resistant rootstock responds to *Fpmd* MR5 stress by upregulating key genes involved in lysine synthesis, leading to an increased secretion of lysine into the rhizosphere. This lysine efflux facilitates the specific recruitment of beneficial *Bacillus*, which in turn antagonizes pathogens like *Fpmd* MR5 and contributes to the maintenance of plant health. We demonstrated through pot and field experiments that the exogenous addition of lysine and *Bacillus* can effectively reshape the rhizosphere microecological environment and alleviate ARD. These findings will help promote the sustainable development of the apple industry. Collectively, our findings reveal a novel mechanism of rhizosphere immune priming mediated by lysine and provide a promising, ecologically friendly strategy for the sustainable management of ARD.

## Methods

Two experiments were performed in this study. In experiment 1, changes in root-associated bacterial community structure and rhizosphere metabolites of CG935 (resistant apple rootstock) and M9T337 (sensitive apple rootstock) under *Fpmd* MR5 stress were studied, and specific microorganisms involved in disease resistance and key rhizosphere metabolites for recruitment of specific microorganisms were isolated and identified. In experiment 2, the effect of combining specific microorganisms with key rhizosphere metabolites on ARD control was examined.

### Experiment 1

#### Test materials and design

Experiment 1 was conducted in Shandong Agricultural University in 2021–2022. The experimental soil was taken from Nanqiu Village, Tai’an City, Shandong Province, China. A wheat–corn rotation was previously used at this site, and there was no history of apple cultivation. The properties of the experimental soil were as follows: soil nitrate nitrogen, 22.5 mg·kg^−1^; ammonium nitrogen, 7.6 mg·kg^−1^; available potassium, 121.1 mg·kg^−1^; available phosphorus, 13.5 mg·kg^−1^; and organic matter, 10.2 g·kg^−1^. M9T337 and CG935 apple tissue culture seedlings were used in experiments; the pathogen *Fpmd* MR5, which causes ARD in China, was previously obtained by our research group and used in experiments. The pot experiment was conducted on November 2, 2021. The experiment was divided into eight treatments: (1) M9T337 planted in normal soil (MZ); (2) inoculation with *Fpmd* MR5 after planting M9T337 in normal soil (MZ_); (3) CG935 planted in normal soil (GZ); (4) inoculation of *Fpmd* MR5 after planting CG935 in normal soil (GZ_); (5) M9T337 planted in sterilized soil (MM); (6) inoculation of *Fpmd* MR5 after planting M9T337 in sterilized soil (MM_); (7) CG935 planted in sterilized soil (GM); and (8) inoculation of *Fpmd* MR5 after planting CG935 in sterilized soil (GM_). *Fpmd* MR5 was inoculated in the MZ_, GZ_, MM_, and GM_ treatments on December 15 and December 22 at a dose of 20 mL per basin and a spore concentration of 1 × 10^7^·mL^−1^. Twenty milliliters of sterile water was added in the MZ, GZ, MM, and GM treatments.

#### Sample collection

On January 2, 2022, sampling was carried out, the seedlings were removed from the pot, the roots were shaken, and the scattered soil (bulk soil) was collected. After the loose soil was removed, the rhizosphere soil from the 1–2 mm roots was brushed off with a sterile brush and collected. After the roots were cleaned, the samples were maintained at − 80 °C.

#### Determination of plant physiological indexes

Plant height and ground diameter were measured using a ruler and Vernier calipers, respectively. The plants were cleaned with water and wiped with toilet paper, and the fresh weight was measured with a balance; the plants were then placed in an oven at 105 °C for 30 min and baked at 80 °C until a constant weight was achieved. The dry weight was measured using a balance. The seedling roots were washed with clean water, placed in a hard plastic box filled with water, and spread out in the water. The professional WinRHIZO (2007 edition) root analysis system was used to process the sample images, and the root length, root surface area, root volume, and number of root tips were recorded. The activity of SOD was determined using the nitroblue tetrazole method, the activity of POD was determined using the guaiacol method, and the activity of CAT was determined using the UV absorption method. The content of MDA was determined using the thiobarbituric acid method [61]. The *C*_i_, *G*_s_, *P*_n_, and *T*_r_ of three healthy functional leaves (the third to fifth unfolded leaves from the top) with the same growth were measured using a CIRAS-3 portable photosynthesis system (PP System, UK) from 9:00 to 11:00 on January 1. The internal light intensity was 450 μmol·m^−2^·s^−1^, the CO_2_ concentration was 360 μL·L^−1^, and the leaf chamber temperature was 25 °C.

#### 16S rRNA gene amplification sequencing

Microbial genomic DNA was extracted from the samples. The extracted DNA was analyzed using 1% agarose gel electrophoresis, and the DNA concentration and purity were determined using a NanoDrop 2000 ultraviolet–visible spectrophotometer (Thermo Scientific, Wilmington, USA). Amplification of the V5–V7 region of the bacterial 16S rRNA was performed with the barcode primer 799 F (5′-AACMGGATTAGATACCCKG-3′)/1193R (5′-ACGTCATCCCCACCTTCC-3′) [62, 63]. The purified amplicon was then sequenced on the Illumina MiSeq PE300 platform/NovaSeq PE250 platform (Illumina, San Diego, USA); paired-end sequencing was performed per the standard protocol of Majorbio Bio-pharma Technology Co., Ltd. (Shanghai, China).

#### Rhizosphere metabolite analysis

To determine the composition of rhizosphere metabolites, the collected samples were sent to Shanghai Meiji Biomedical Technology Co., Ltd., for non-targeted metabolome sequencing analysis. The samples were analyzed by LC–MS/MS using Thermo Field’s ultra-high-performance liquid chromatography-tandem Fourier transform mass spectrometry UHPLC-Q Exactive HF-X system. The specific data can be found in the Supplementary Table 10.

#### Isolation and identification of strains

Ten grams of rhizosphere soil was placed in 250 mL of an aseptic triangle bottle containing 90 mL of aseptic water and aseptic glass beads. It was then shaken at 180 r·min^−1^ on a shaking table at 28 °C for 20 min. After that, 1 mL of supernatant was added to a test tube containing 9 mL of aseptic water. After vortexing, the rhizosphere soil suspension diluted by 10^−2^ was obtained. Soil suspensions were successively diluted to 10^−7^ via the gradient dilution method, and rhizosphere soil suspensions diluted to different degrees were obtained. TSA, R2A, and LB solid media were diluted to prepare 0.01, 0.1, 0.2, 0.5, and 1 × TSA, R2A, and LB-coated media, respectively. Rhizosphere soil suspensions with concentrations of 100 μL, 10^−5^, 10^−6^, and 10^−7^ were coated on 0.01, 0.1, 0.2, 0.5, and 1 × TSA, R2A, and LB-coated media. Colonies with different morphological characteristics were selected and purified by the plate marking method. They were then stored in a refrigerator at − 80 °C. Bacterial 16S universal primers 27 F (5′-AGAGTTTGATCCTGGCTCAG-3′) and 1492R (5′-GGTTACCTTGTT ACGACTT-3′) were used to amplify the 16S rRNA gene sequence. The 50 μL amplification system was as follows: 25 μL of PCR Master Mix (Thermo Fisher Technologies), 2 μL of forward primer 27 F (10 μmol·L^−1^), 2 μL of reverse primer 1492R (10 μmol·L^−1^), 2 μL of DNA template, and 19 μL of sterile water. The PCR amplification conditions were as follows: 95 °C for 3 min; 30 cycles of 95 °C for 30 s, 55 °C for 30 s, and 72 °C for 1 min; and 72 °C for 10 min. The amplified products were sent to Shenggong Bioengineering (Shanghai) Co., Ltd., for sequencing, and the sequencing results were analyzed by NCBI for homology comparison; phylogenetic analysis was performed using MEGA 5 software, and phylogenetic trees were constructed using the neighbor-joining algorithm and Jukes-Cantor model.

#### Qualitative analysis of the functions of rhizosphere bacteria

The strains were inoculated into phosphorus-solubilizing medium, iron carrier medium, amylase diastase medium, protease medium, cellulase medium, ammonia production detection medium, and IAA production detection medium, and the related functions were qualitatively analyzed [64].

#### Evaluation of the antagonistic ability of rhizosphere bacteria

A hole punch was used to collect bacteria from the edge of the pathogen (*F. proliferatum*, *F. moniliforme*, *F. oxysporum*, *F. solani*, and *F. proliferatum* f*.* sp *malus domestica* (*Fpmd* MR5)) medium, and the bacteria were inoculated into the center of the PDA medium with the inoculation ring. The isolated bacteria were inoculated in the upper, lower, left, and right directions of the pathogen *Fusarium* and cultured in a constant temperature incubator at 28 °C for 5–7 days. The inhibition ability of strain R29 on the pathogen *Fusarium* was calculated. To determine whether strains had antibiotic synthesis genes, primers in Supplementary Table 7 were used to amplify genes associated with the synthesis of bacillomycin, iturins, subtilisin, fengycins, and surfactins, and PCR products were detected by agarose gel electrophoresis [39]. To determine the influence of the strain fermentation solution on the germination of pathogen *Fusarium* spores, a sterilized concave slide was placed in a sterile Petri dish, 50 µL of spore suspension was absorbed and added to the concave slide, then 50 μL strain R29 fermentation filtrate was added, 50 μL sterile water was added to the control treatment, and the Petri dish was sealed and cultured at 28 °C for 12 h. Spore germination was observed under a microscope, and each treatment was repeated three times. To determine the inhibition rate of the strain fermentation solution against *Fusarium*, the strain was inoculated into LB liquid medium and placed in a constant temperature shaking table at 37 °C for 24 h to obtain the fermentation solution. The fermentation solution was filtered and added to PDA solid medium; it was then cooled to approximately 50 °C, and the pathogen *Fusarium* was inoculated. To determine the effect of the strain fermentation solution on the mycelial morphology of the *Fpmd* MR5, the prepared fermentation solution was added to the culture dish inoculated with the *Fpmd* MR5 and treated for 1 h; treatment with sterile water was used as a control. The treated mycelia were collected with sterile tweezers, placed in a fixative containing 2.5% glutaraldehyde, and observed under a scanning electron microscope.

#### Determination of the pathogen-inhibiting substance in the fermentation solution of strain R29

To determine the substance inhibiting the growth of pathogenic *Fusarium* in the strain fermentation solution, the fermentation solution was combined with ethyl acetate along with a 1:1 (v/v) ratio of the extract; anhydrous sodium sulfate was added to absorb excess water, and an EYELA rotating evaporation instrument was used for spin evaporation under 42 °C and 0.05 MPa. Ethyl acetate was then added and dissolved; the solution was filtered through a 0.22-μm Nylon66 microporous membrane. This was followed by ultrasonic treatment for 10 min. The capillary column was Rtx-5MS (60.0 m × 0.25 μm × 0.25 mm), the inlet temperature was 250 °C, and the capillary column was RTX-5 ms. The carrier gas was high-purity helium, the flow control mode was pressure (117.6 Kpa), the total flow rate was 14.0 mL·min^−1^, the column flow rate was 1.00 mL·min^−1^, the line velocity was 25.6 cm·s^−1^, and the purge flow rate was 3.0 mL·min^−1^. After incubation at 50 °C for 2 min, the temperature was increased from 10 to 180 °C over 1 min. It was then heated to 300 °C at 6 °C·min^−1^ and kept warm for 5 min. The ion source temperature was 200 °C, and the interface temperature was 280 °C. Data were acquired via Q3 scanning, the solvent delay time was 3 min, the charge-mass ratio was 45–650 m·z^−1^, and the possible structure of the substance was determined according to the spectral database NIST17. The specific data can be found in the Supplementary Tables 11 and 12. The ability of the identified substances to inhibit the pathogen *Fusarium* was tested.

#### Protective effect of strain R29 on plant roots

The roots of M9T337 seedlings were subjected to different treatments (sterile water treatment for 24 h, sterile water treatment for 12 h + *Fpmd* MR5 spore suspension treatment for 12 h, and strain R29 fermentation solution treatment for 12 h + *Fpmd* MR5 spore suspension treatment for 12 h), and the treated roots were placed in 50% FAA fixed solution. Paraffin sections and periodic acid-Schiff staining were performed [39].

#### Evaluation of the chemotactic effect and utilization of lysine by strain R29

Chemotaxis was measured by titration. The bacteria were cultured in BPM liquid medium for 1 day and then removed for use. The supernatant was discarded after centrifugation at 4 °C and 8000 r min^−1^ for 5 min. The bacteria were washed twice with the chemotactic buffer pre-cooled with a refrigerator at 4 ℃. When the mineral salt medium was cooled to approximately 40 °C, bacterial solution was added until an OD600 of 0.6 was achieved, and the contents of the plate were immediately decanted. After the plate was cooled, 10 μL of lysine was added to the center of the plate. The lysine concentration was 100 mmol·L^−1^, and the plate was cultured in an incubator at 28 °C. The chemoattractant reaction was observed within 2–6 h and photographed. Lysine and strain R29 were cultured in medium containing 1/10 TSB, the lysine concentration was 100 μmol·L^−1^, and the initial OD600 of strain was 0.05. OD600 of the bacterial solution was determined after incubation at 37 °C and 180 r·min^−1^ for 12 h [65]. The composition of the culture medium is shown in Supplementary Table 9.

### Experiment 2

#### Test materials and design

##### Greenhouse pot experiment

Experiment 2 was conducted at Shandong Agricultural University in December 2023; the soil was collected from 25-year-old apple orchards in Tanqingwan Village, Tai’an City, Shandong Province, China. The properties of the soil were as follows: soil nitrate nitrogen, 20.4 mg·kg^−1^; ammonium nitrogen, 5.4 mg·kg^−1^; available potassium, 76.4 mg·kg^−1^; available phosphorus, 8.9 mg·kg^−1^; and organic matter, 8.4 g·kg^−1^. M9T337 apple tissue culture seedlings were used in this experiment. Four treatments were performed in this experiment: replant control, lysine, strain R29, and lysine + strain R29. The M9T337 tissue culture seedlings were transplanted into a pot containing old apple orchard soil, and the corresponding treatment was performed under the same management procedures.

##### Open-field pot experiment

This experiment was conducted in Shandong Agricultural University in April 2023, and the soil tested was the same as that in the greenhouse pot experiment. The apple seedlings tested were *M*. *hupehensis* Rehd., a commonly used apple rootstock in China. The experiment was performed in the same location as the greenhouse pot experiment.

##### Field experiment

The field experiment was conducted in April 2024 in Muping, Shandong, China, and Haiyang, Shandong, China. The previous crops in both locations were apple trees that had been planted for 30 years. The properties of the Muping soil were as follows: nitrate nitrogen, 18.5 mg·kg^−1^; ammonium nitrogen, 4.3 mg·kg^−1^; available potassium, 45.6 mg·kg^−1^; available phosphorus, 8.3 mg·kg^−1^; and organic matter, 8.7 g·kg^−1^. The properties of the Haiyang soil were as follows: nitrate nitrogen, 44.2 mg·kg^−1^; ammonium nitrogen, 9.6 mg·kg^−1^; available potassium, 92.6 mg·kg^−1^; available phosphorus, 17.4 mg·kg^−1^; and organic matter, 10.3 g·kg^−1^. This experiment was performed at the same site as the greenhouse pot experiment. Apple saplings were planted in April 2024. The rootstock of the apple saplings at Muping was M9T337, and the scion was Yanfei. The rootstock of apple saplings at Haiyang was M9T337, and the scion was Yanfu 8.

### Determination of the antioxidant capacity of plants

The leaves and roots of the plants were soaked with 0.2% nitro blue tetrazolium (NBT) dyeing solution, bathed in water at 37 °C for 2 h, decolorized with 80% ethanol at 80 °C, rinsed, and photographed under a microscope [66]. Staining and microscopy were performed according to the instructions of the 3, 3′-diaminobenzidine (DAB) kit (20 ×, DA1010, Solarbio) [67].

### Soil enzyme activity and microbial population determination

Soil phosphatase was determined using the phenylene disodium phosphate colorimetric method, urease colorimetric method, sucrase colorimetric method, and catalase volumetric method [68]. Soil culturable bacteria, fungi, and actinomycetes were all determined using the dilution plate counting method [69]. The CFX Connect system (BIO-RAD, Hercules, CA, USA) was used for fluorescence quantitative analysis of the pathogen *Fusarium* in soil. Information on primers, reaction systems, and thermal procedures is shown in Supplementary Table 8. The 16S rRNA gene amplification sequence was completed by Shanghai Meiji Biomedical Technology Co., Ltd., and the primer was 338 F (5′-ACTCCTACGGGAGGCAGCAG-3′)/806R (5′-GGACTACHVGGGTWTCTAAT-3′).

### Statistical analyses

UPARSE (version 7.0.1090) was used to cluster processed sequences into operational taxa (OTU) with a 97% similarity threshold [70]. The SILVA database (version 138/16S-bacteria database) was classified and analyzed using the RDP Classifier algorithm. The confidence threshold was 70%. PICRUSt2.2.0 was used to predict potential functional changes in bacterial communities in soil samples. α diversity was statistically analyzed using Shannon and Simpson indices (Mothur.1.30.1 version). Collinear network analysis was performed using R software (version 4.1.1) and Gephi (version 0.10). KEGG pathway enrichment analysis, microbiome and metabolite correlation analysis, and differential metabolite analysis were performed using Python (version 1.0.0) and R software. SPSS software (version 19.0, SPSS Inc., Chicago, USA) was used to calculate the mean and standard error of the mean. Plots were made using GraphPad Prism 8.0 (San Diego, CA 92108, USA). Student’s *t*-test was used to assess the significance of differences between treatments.

Structural equation modeling was used to evaluate the direct and indirect relationships between the relative abundance of OTU5381, the relative abundance of the lysine degradation function, the Simpson index, soil enzyme activity, the number of *Fusarium*, *G*_s_, and plant growth. The data were processed before modeling. The distribution and normality of all endogenous variables were tested. The parameters of the model were fitted, and the overall goodness of fit was tested. The following information was used for fitting: probability level (*P* > 0.05), Chi-square degree of freedom ratio (*χ*^2^/df < 3), comparative fitting index (CFI > 0.95), value-added fitting index (IFI > 0.9), Benteler-Burnet gauge index (NFI > 0.9), and the square root of approximation error (RMSEA < 0.05).

To complement the null hypothesis significance testing and provide a measure of biological relevance, the effect size was calculated as Cohen’s *d* for *t*-tests (or *η*^2^ (eta-squared) for ANOVAs). The magnitude of the effect size was interpreted as follows: |d| ≈ 0.2, small; |d| ≈ 0.5, medium; |d| ≈ 0.8, large. Furthermore, a post hoc power analysis was conducted using G*Power software (version 3.1.9.7) to determine the statistical power (1—β) of the tests given the observed effect sizes, sample size (*n* = 5 per group), and *α* level of 0.05. This analysis informs the probability that our experimental design could correctly detect an existing effect.

## Supplementary Information

Supplementary Material 1.

Supplementary Material 2.

Supplementary Material 3.

Supplementary Material 4.

## Data Availability

The raw data of the 16S rRNA gene microbiome sequence are available in the NCBI SRA repository as Bio Projects PRJNA1255861. The metabolomics data have been deposited to MetaboLights repository with the study identifier MTBLS12530 (https://www.ebi.ac.uk/metabolights/reviewer761adc54-ff69-4f7a-9aaa-3487fc9cfa0e).
