# Supplementary material for: From metabolic fingerprints to field solutions: engineering the apple rhizosphere microbiome via host-directed Bacillus recruitment for sustainable apple replant disease control
Source: Microbiome. 2025 Dec 23;14:43. doi: 10.1186/s40168-025-02301-9 (PMC12836847; doi:10.1186/s40168-025-02301-9)
Supplement: Supplementary file 2 — Supplementary Material 1. [file 40168_2025_2301_MOESM1_ESM.docx]

**Supplementary for**

Weitao Jiang^1,†^, Ran Chen^1,†^, Lefen Song^1^, Lei Qin^1^, Xin Xu^1^, Xiaoxuan Li^1^, Lei Zhao^1^, Jinhui Lyu^1^, Xiaoqi Wang^1^, Gongshuai Wang^3^, Xuesen Chen^1^, Yusong Liu^1^, Mei Wang^1^, Chengmiao Yin^1*^, Yanfang Wang^2*^, Zhiquan Mao^1*^

^1^ College of Horticulture Science and Engineering Shandong Agricultural University Tai’an, Shandong, 271018, China

^2^ College of Chemistry and Material Science Shandong Agricultural University Tai’an, Shandong, 271018, China

^3^ College of Forestry Engineering Shandong Agriculture and Engineering University, Ji’nan, Shandong, 250000, China

^†^Weitao Jiang and Ran Chen contributed equally to this work.

^*^Correspondence: Chengmiao Yin [yytxyinchengmiao@163.com;](mailto:yytxyinchengmiao@163.com;) Yanfang Wang [wyanfang@sdau.edu.cn;](mailto:wyanfang@sdau.edu.cn;) Zhiquan Mao [zhiquanmao@1](mailto:mzhiquan@sdau.edu.cn)63.com.

**Metabolite Extraction**

Sample processing：

1000 mg soil sample was added to a 2 mL centrifuge tube and a 6 mm diameter grinding bead was added. 400 μL of extraction solution (methanol: water = 4:1 (v:v) containing four internal standards ( 0.02 mg·mL^-1^ L-2-chlorophenylalanine, etc.) were used for metabolite extraction. Samples were ground by the Wonbio-96c ( Shanghai wanbo biotechnology co., LTD) frozen tissue grinder for 6 min (-10°C, 50 Hz), followed by low-temperature ultrasonic extraction for 30 min (5°C, 40 kHz). The samples were left at -20°C for 30 min, centrifuged for 15 min (4°C, 13000 g), and the supernatant was transferred to the injection vial for LC-MS/MS analysis. Additionally, 20 μL of the supernatant were separately taken from each sample, and then mixed together to serve as the quality control sample.

**(UPLC-MS/MS) analysis**

The LC-MS/MS analysis of sample was conducted on a UHPLC-Q Exactive HF-X system equipped with an ACQUITY HSS T3 column (100 mm × 2.1 mm i.d., 1.8 μm; Waters, USA) at Majorbio Bio-Pharm Technology Co. Ltd. (Shanghai, China). The mobile phases consisted of 0.1% formic acid in water:acetonitrile (95:5, v/v) (solvent A) and 0.1% formic acid in acetonitrile: isopropanol: water (47.5:47.5:5, v/v/v) (solvent B). The flow rate was 0.4 mL·min^-1^ and the column temperature was 40°C.

MS conditions:

The UPLC system was coupled to a UHPLC-Q Exactive HF-X system Mass Spectrometer equipped with an electrospray ionization (ESI) source operating in positive mode and negative mode. The optimal conditions were set as followed: source temperature at 400℃ ; sheath gas flow rate at 50 arb; Aux gas flow rate at 13 arb; ion-spray voltage floating (ISVF) at -3500V in negative mode and 3500V in positive mode, respectively; Normalized collision energy, 20-40-60V rolling for MS/MS. Data acquisition was performed with the Data Dependent Acquisition (DDA) mode. The detection was carried out over a mass range of 70-1050 m/z.

**
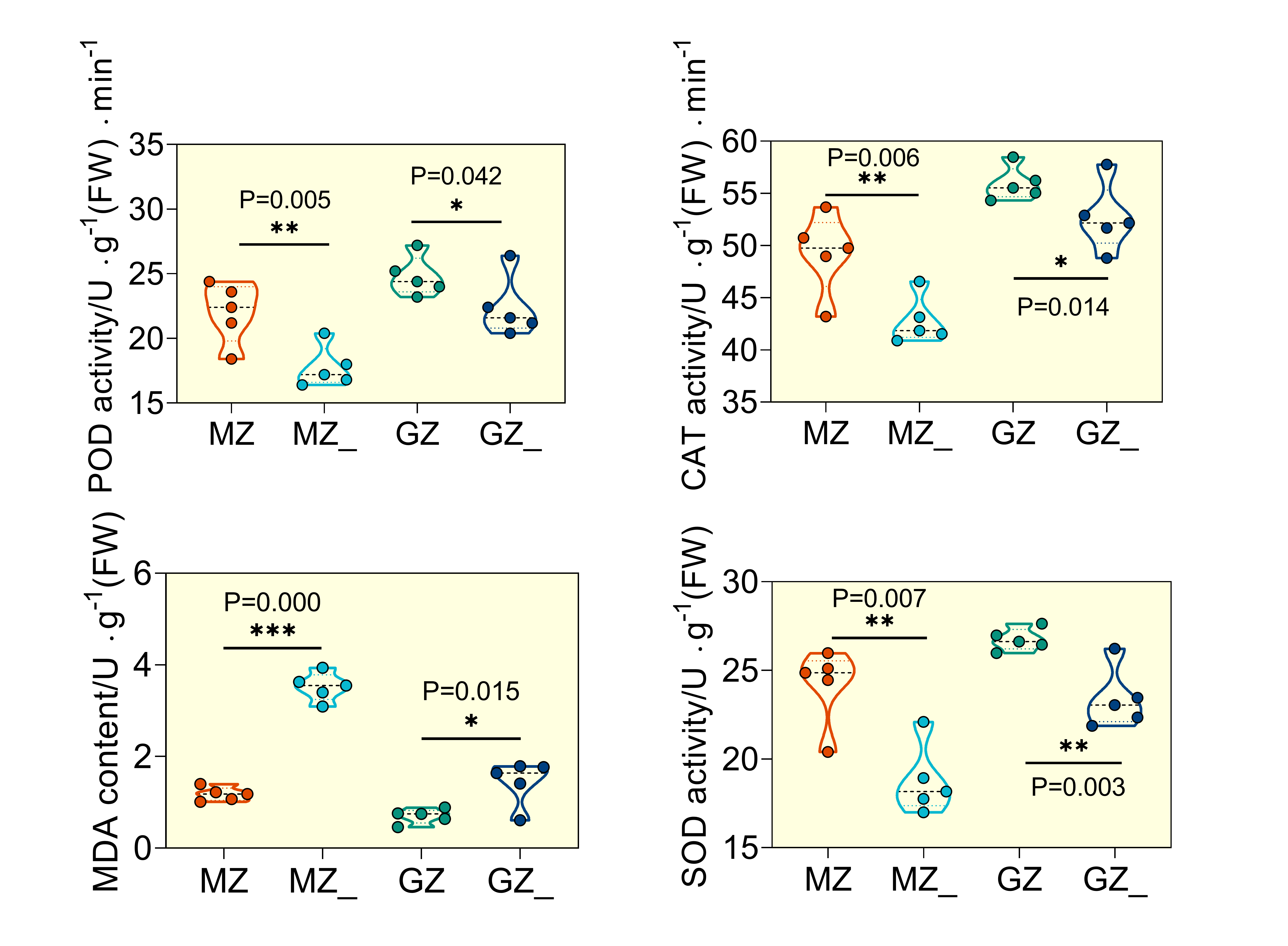
**

**Supplementary Figure 1. Effects of *Fpmd* MR5 on protective enzyme activity and malondialdehyde content in roots of CG935 and M9T337.** The asterisk on the bars indicates significant differences between the two groups as measured via a two-tailed Student’s *t*-test (*0.01<P≤0.05, **0.001<P≤0.01, ***P≤0.001). CG935: Resistant rootstock; M9T337: Sensitive rootstock. MZ: M9T337 planted in normal soil; MZ_ : Inoculated with *Fpmd* MR5 after planting M9T337 in normal soil; GZ: CG935 planted in normal soil; GZ_ : Inoculated with *Fpmd* MR5 after planting CG935 in normal soil.

**
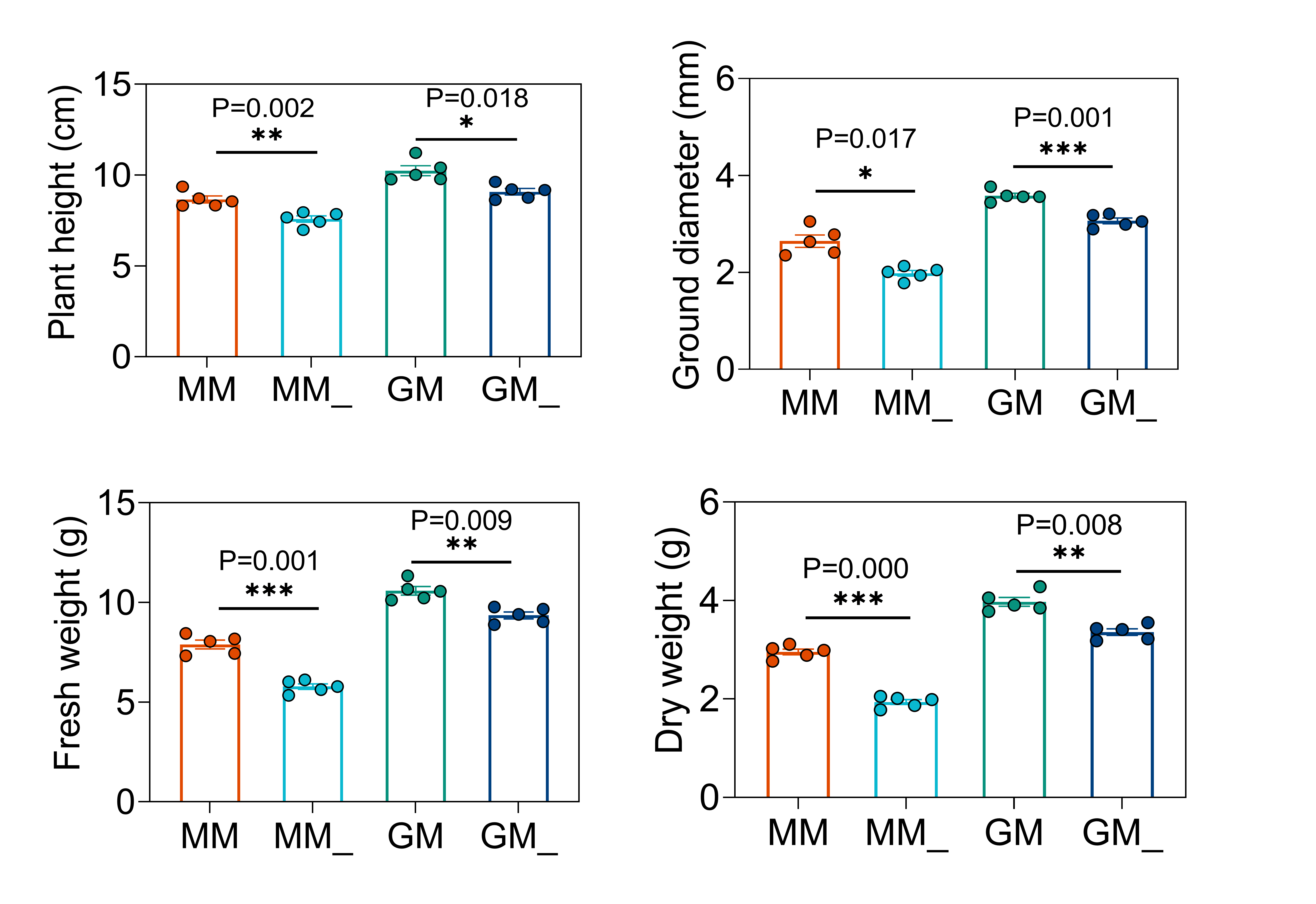
**

**Supplementary Figure 2. Effects of *Fpmd* MR5 on the growth of CG935 and M9T337.** The asterisk on the bars indicates significant differences between the two groups as measured via a two-tailed Student’s *t*-test (*0.01<P≤0.05, **0.001<P≤0.01, ***P≤0.001) and the mean±SEM (n=5) for each histogram. CG935: Resistant rootstock; M9T337: Sensitive rootstock. MM: M9T337 planted in sterilized soil; MM_ : Inoculated with *Fpmd* MR5 after planting M9T337 in sterilized soil; GM: CG935 planted in sterilized soil; GM_ : Inoculated with *Fpmd* MR5 after planting CG935 in sterilized soil.


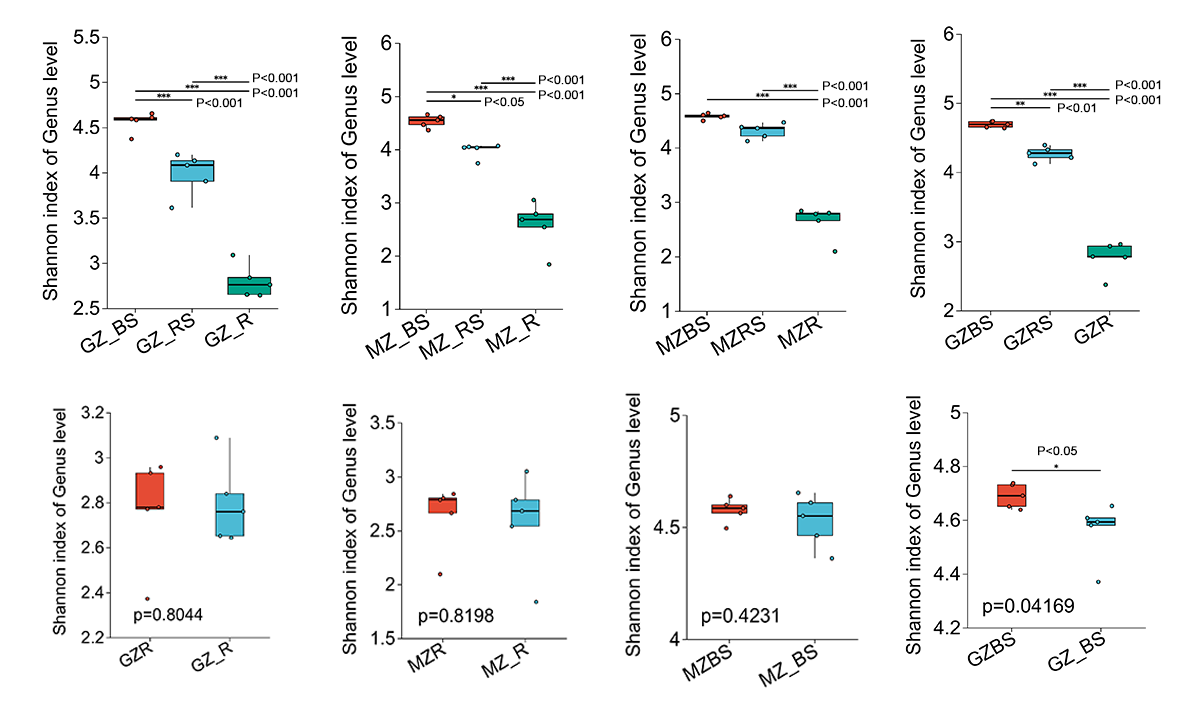


**Supplementary Figure 3.** **Shannon index of bulk soil and root bacterial communities in CG935 and M9T337 under *Fpmd* MR5 stress.** The asterisk on the bars indicates significant differences between the two groups as measured via a two-tailed Student’s *t*-test (*0.01<P≤0.05, **0.001<P≤0.01, ***P≤0.001). MZBS: MZ-treated bulk soil; MZ_BS: MZ_-treated bulk soil; GZBS: GZ-treated bulk soil; GZ_BS: GZ_-treated bulk soil；MZR: MZ-treated root; MZ_R: MZ_-treated root; GZR: GZ-treated root; GZ_R: GZ_-treated root.

**
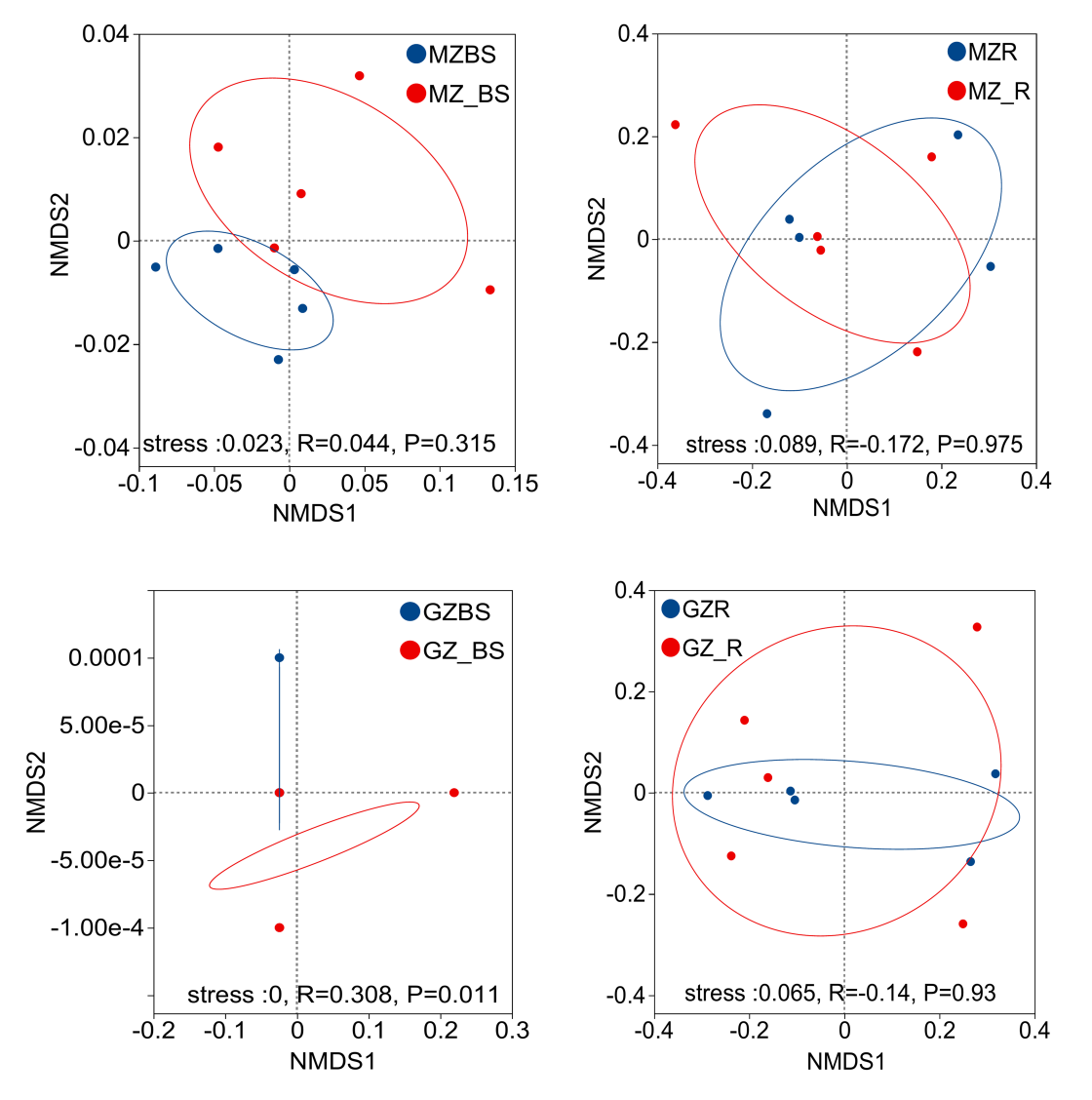
**

**Supplementary Figure 4. β diversity of rhizosphere bacterial communities in CG935 and M9T337 under *Fpmd* MR5 stress.** MZBS: MZ-treated bulk soil; MZ_BS: MZ_-treated bulk soil; GZBS: GZ-treated bulk soil; GZ_BS: GZ_-treated bulk soil；MZR: MZ-treated root; MZ_R: MZ_-treated root; GZR: GZ-treated root; GZ_R: GZ_-treated root.

**
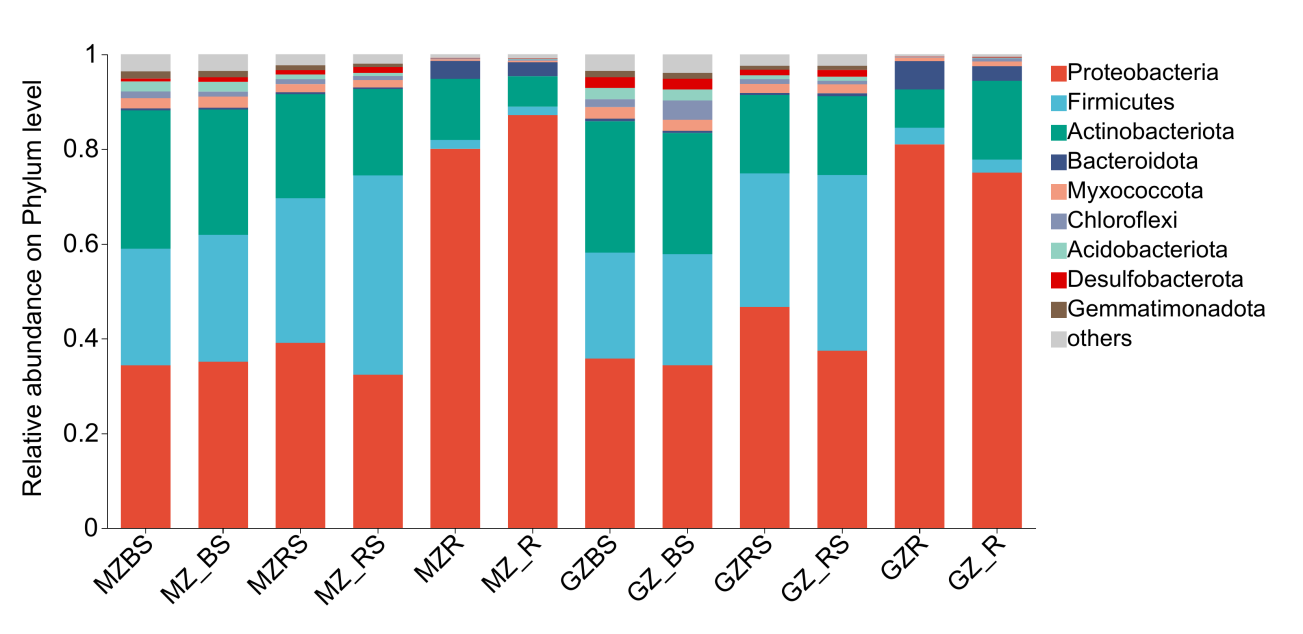
**

**a**

**
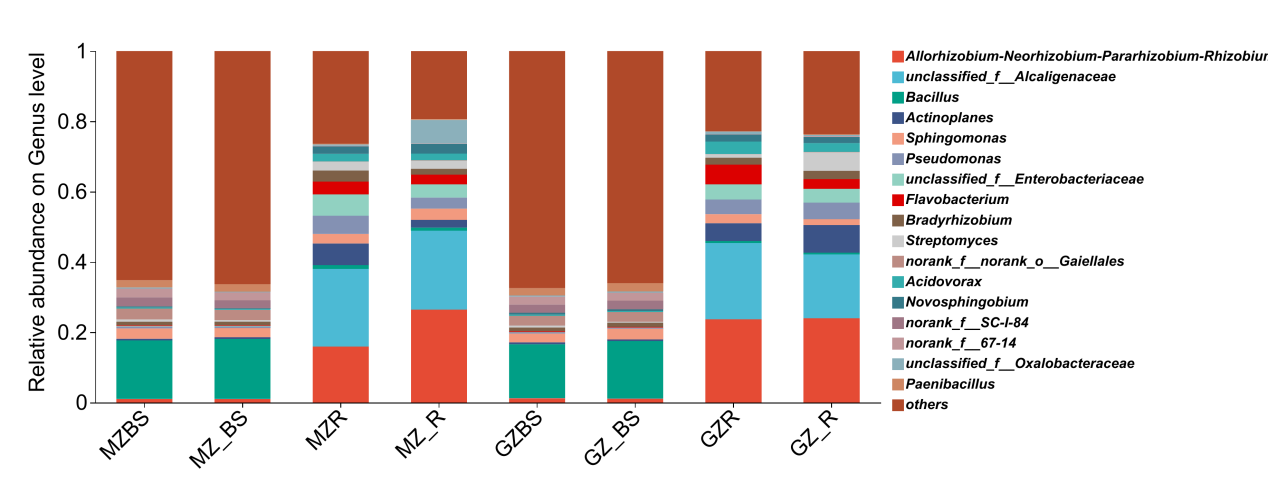
**

**Supplementary Figure 5. Changes in root-associated bacterial community structure of CG935 and M9T337 under *Fpmd* MR5 stress.** MZBS: MZ-treated bulk soil; MZ_BS: MZ_-treated bulk soil; GZBS: GZ-treated bulk soil; GZ_BS: GZ_-treated bulk soil; MZRS: MZ-treated rhizosphere soil; MZ_RS: MZ_-treated rhizosphere soil; GZRS: GZ-treated rhizosphere soil; GZ_RS: GZ_-treated rhizosphere soil; MZR: MZ-treated root; MZ_R: MZ_-treated root; GZR: GZ-treated root; GZ_R: GZ_-treated root.


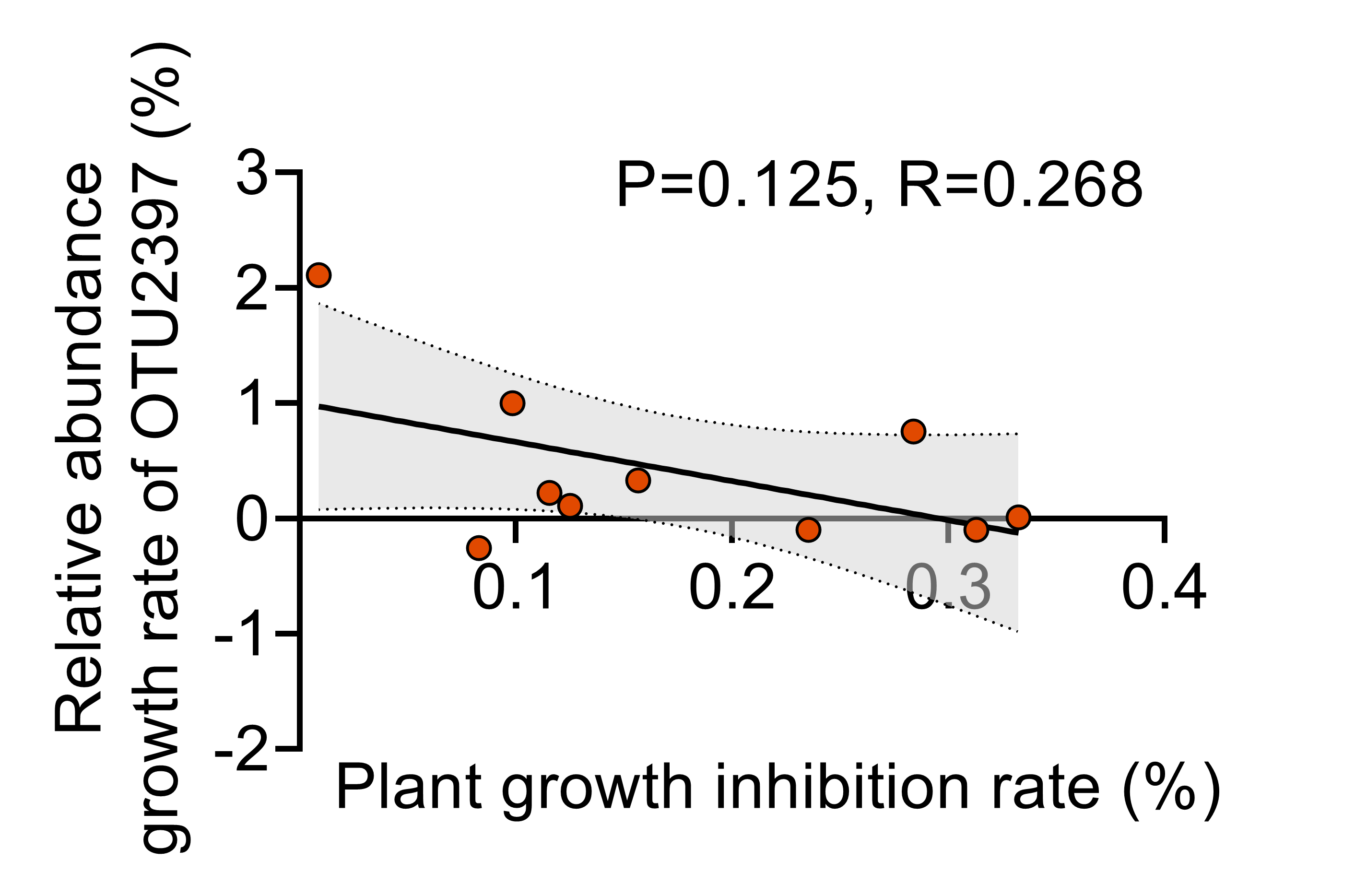

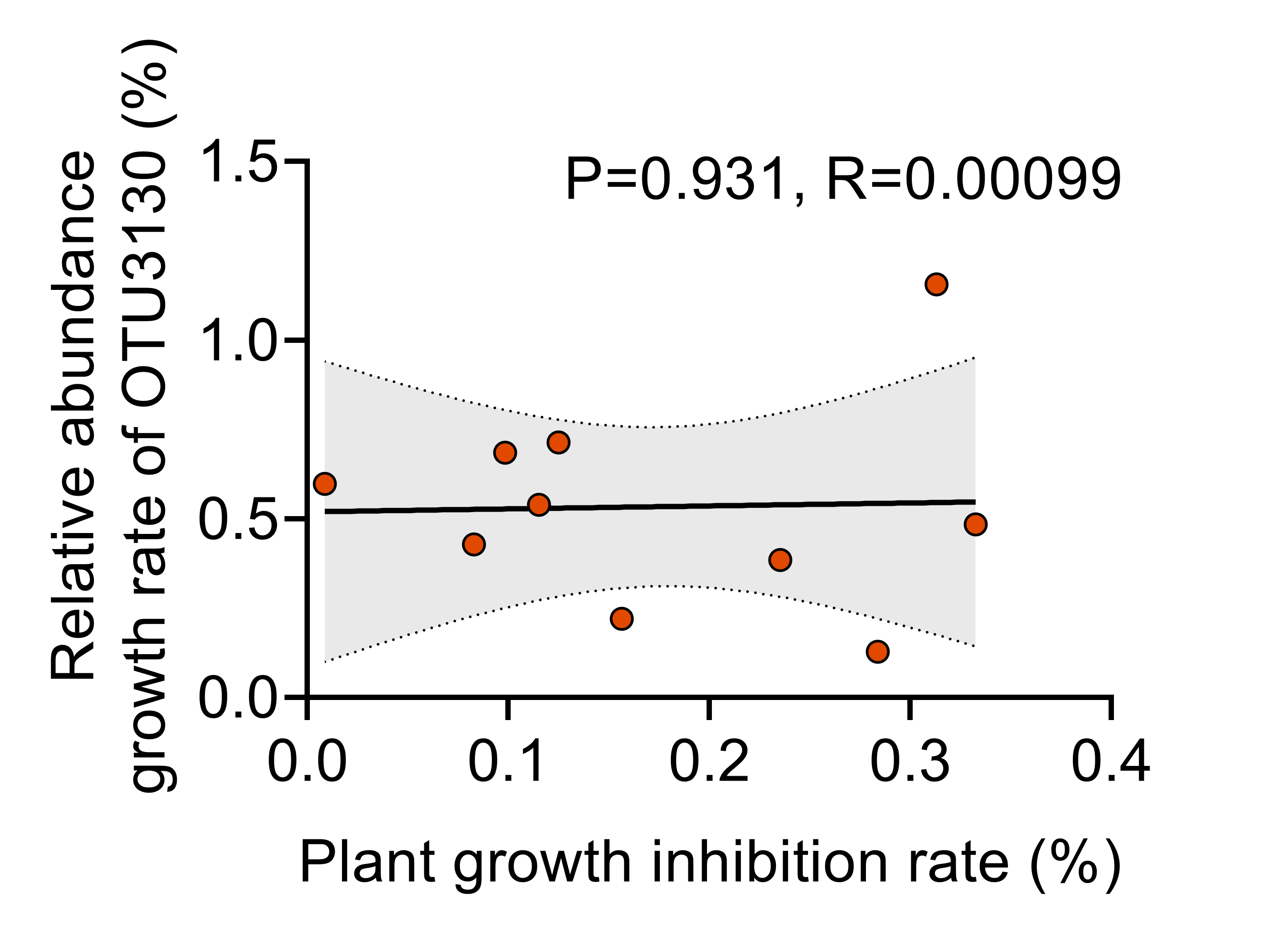


**Supplementary Figure 6. Correlation analysis between the relative abundance of OTU3130 and OTU2397 and the dry weight inhibition rate of plants.**

**
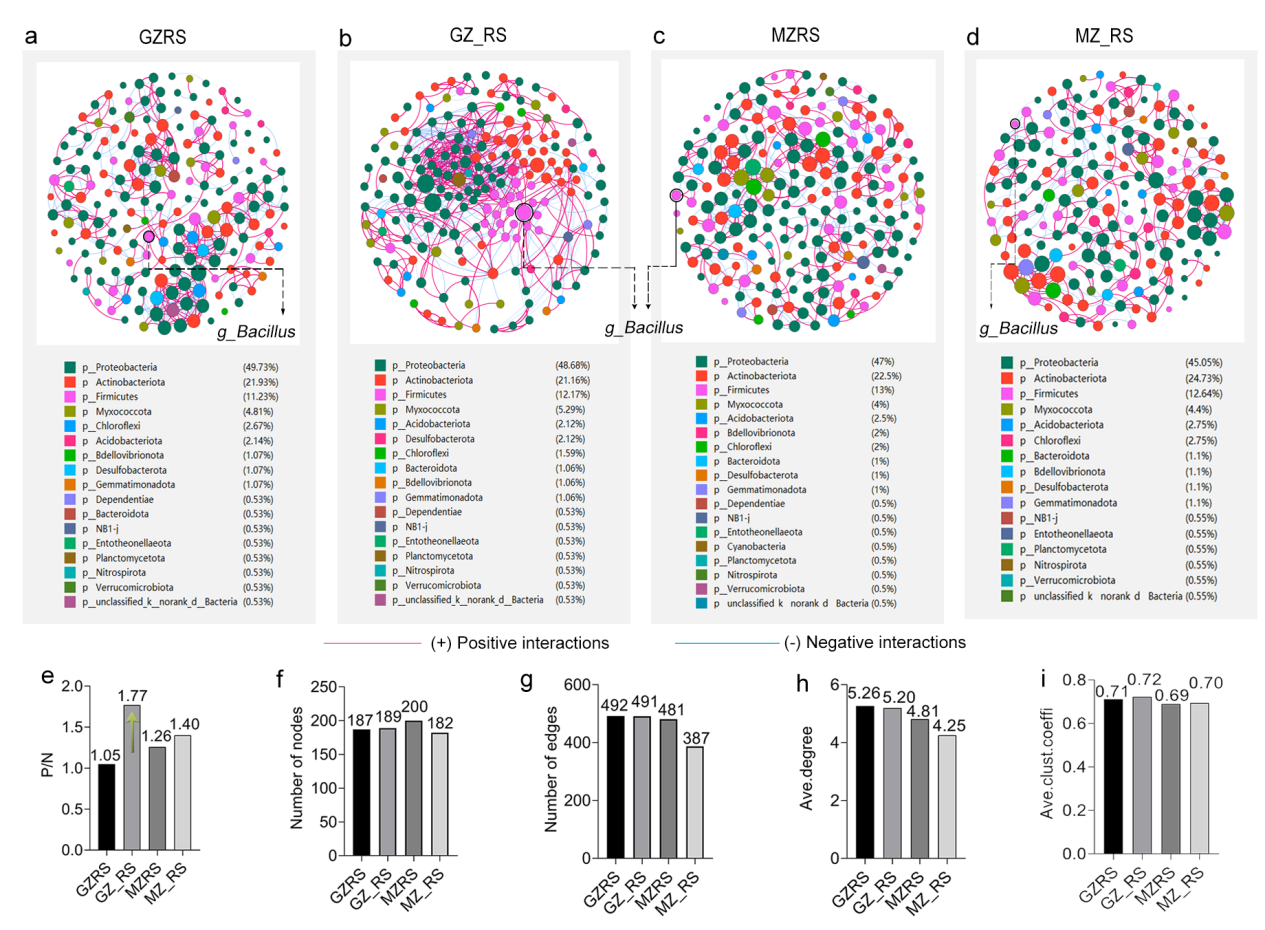
**

**Supplementary Figure 7. Changes in the rhizosphere bacterial community structure-collinear network of CG935 and M9T337 under *Fpmd* MR5 stress.** **a** GZRS bacterial collinearity network; **b** GZ_RS bacterial collinearity network; **c** MZRS bacterial collinearity network **d** MZ_RS bacterial collinearity network; **e** Positive and negative correlation ratio of bacterial species; **f** Number of bacterial nodes; **g** Number of bacterial edges; **h** Ave. degree; **i** Ave. cluster. coeffi. MZRS: MZ-treated rhizosphere soil; MZ_RS: MZ_-treated rhizosphere soil; GZRS: GZ-treated rhizosphere soil; GZ_RS: GZ_-treated rhizosphere soil.

**
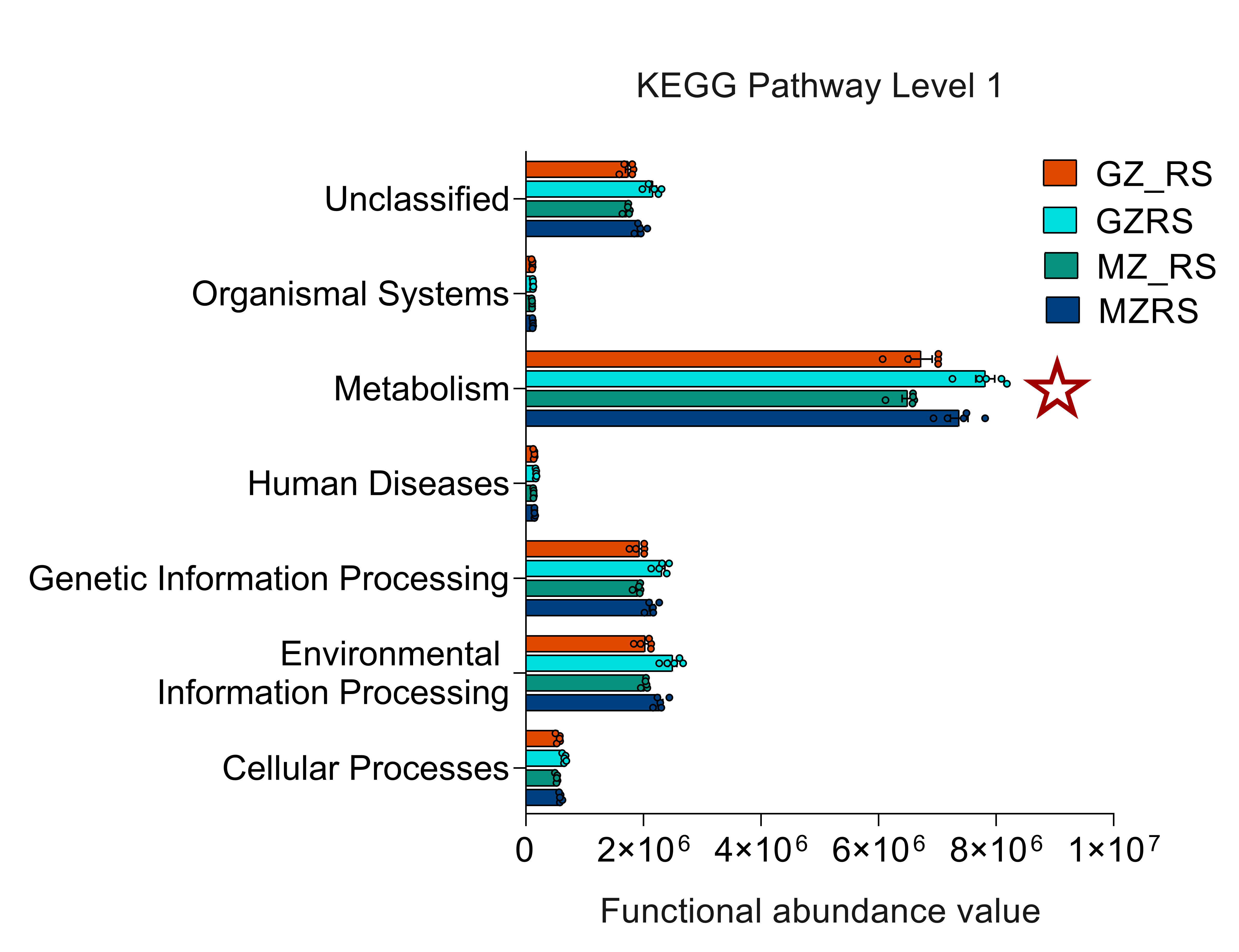

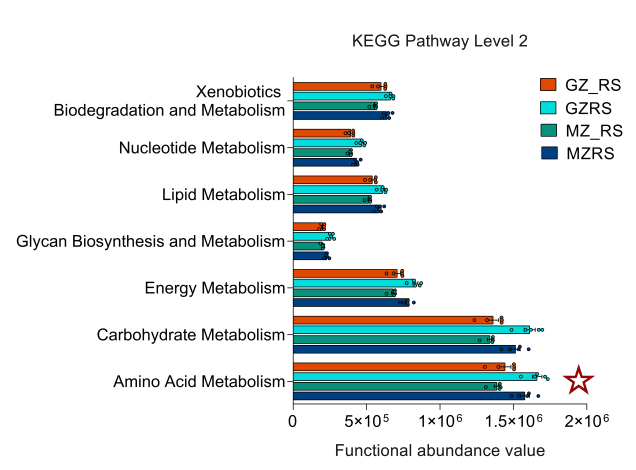
**

b

a

**Supplementary Figure 8. Changes in the rhizosphere bacterial community function of CG935 and M9T337 under *Fpmd* MR5 stress.** **a** The functional abundance values of the first level KEGG pathway in GZ_RS, GZRS, MZ_RS, and MZRS; **b** The functional abundance values of the second level KEGG pathway in GZ_RS, GZRS, MZ_RS, and MZRS. MZRS: MZ-treated rhizosphere soil; MZ_RS: MZ_-treated rhizosphere soil; GZRS: GZ-treated rhizosphere soil; GZ_RS: GZ_-treated rhizosphere soil.


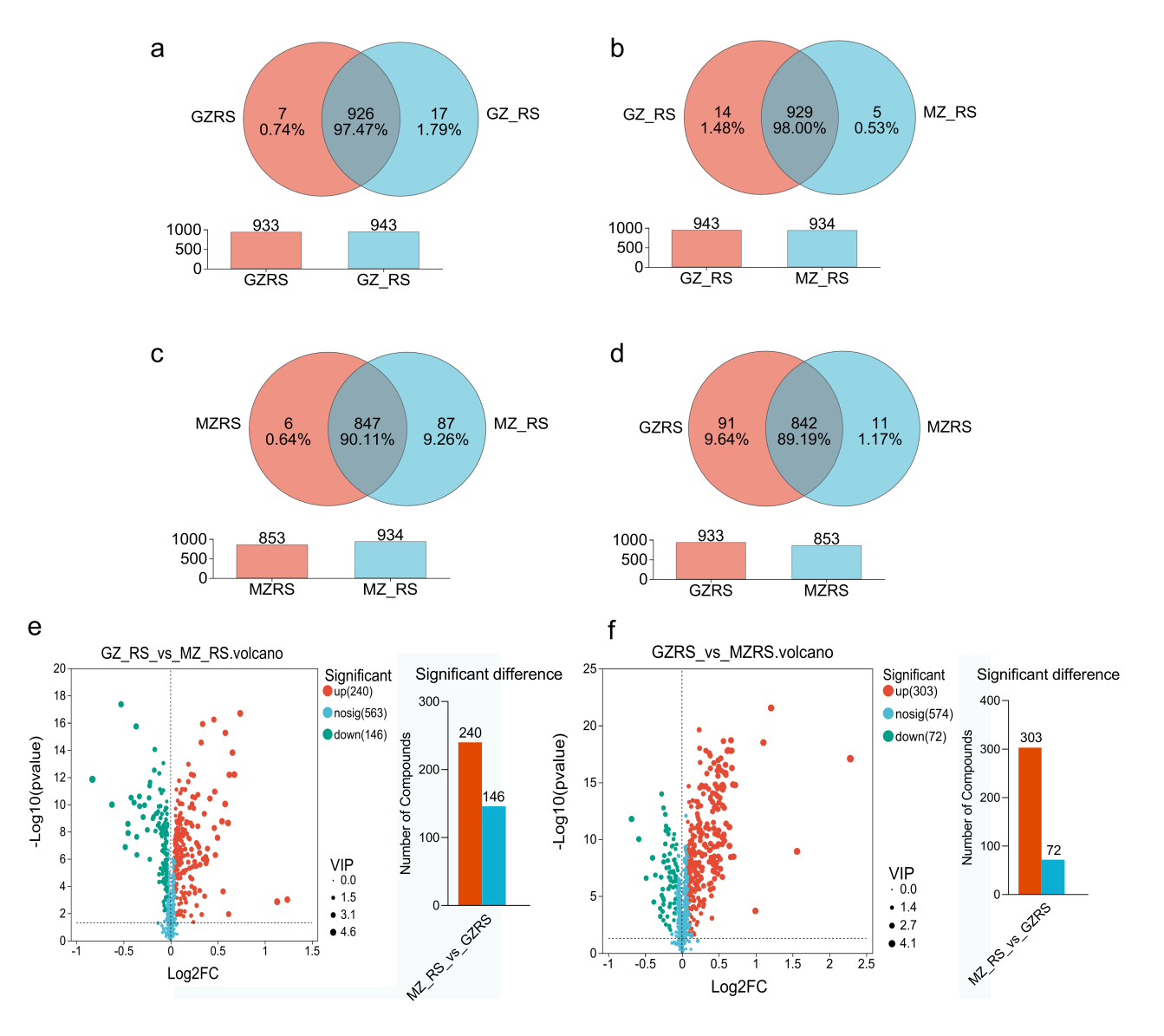


**Supplementary Figure 9. Statistical difference of rhizosphere metabolites between CG935 and M9T337 under *Fpmd* MR5 stress.** **a** The quantity of rhizosphere metabolisms unique or shared by GZRS and GZ_RS; **b** The quantity of rhizosphere metabolisms unique or shared by GZ_RS and MZ_RS; **c** The quantity of rhizosphere metabolisms unique or shared by MZRS and MZ_RS; **d** The quantity of rhizosphere metabolites unique or shared by GZRS and MZRS; **e** Intergroup differential metabolism statistics of GZ_RS and MZ_RS; **f** Intergroup differential metabolism statistics of GZRS and MZRS. MZRS: MZ-treated rhizosphere soil; MZ_RS: MZ_-treated rhizosphere soil; GZRS: GZ-treated rhizosphere soil; GZ_RS: GZ_-treated rhizosphere soil.

**
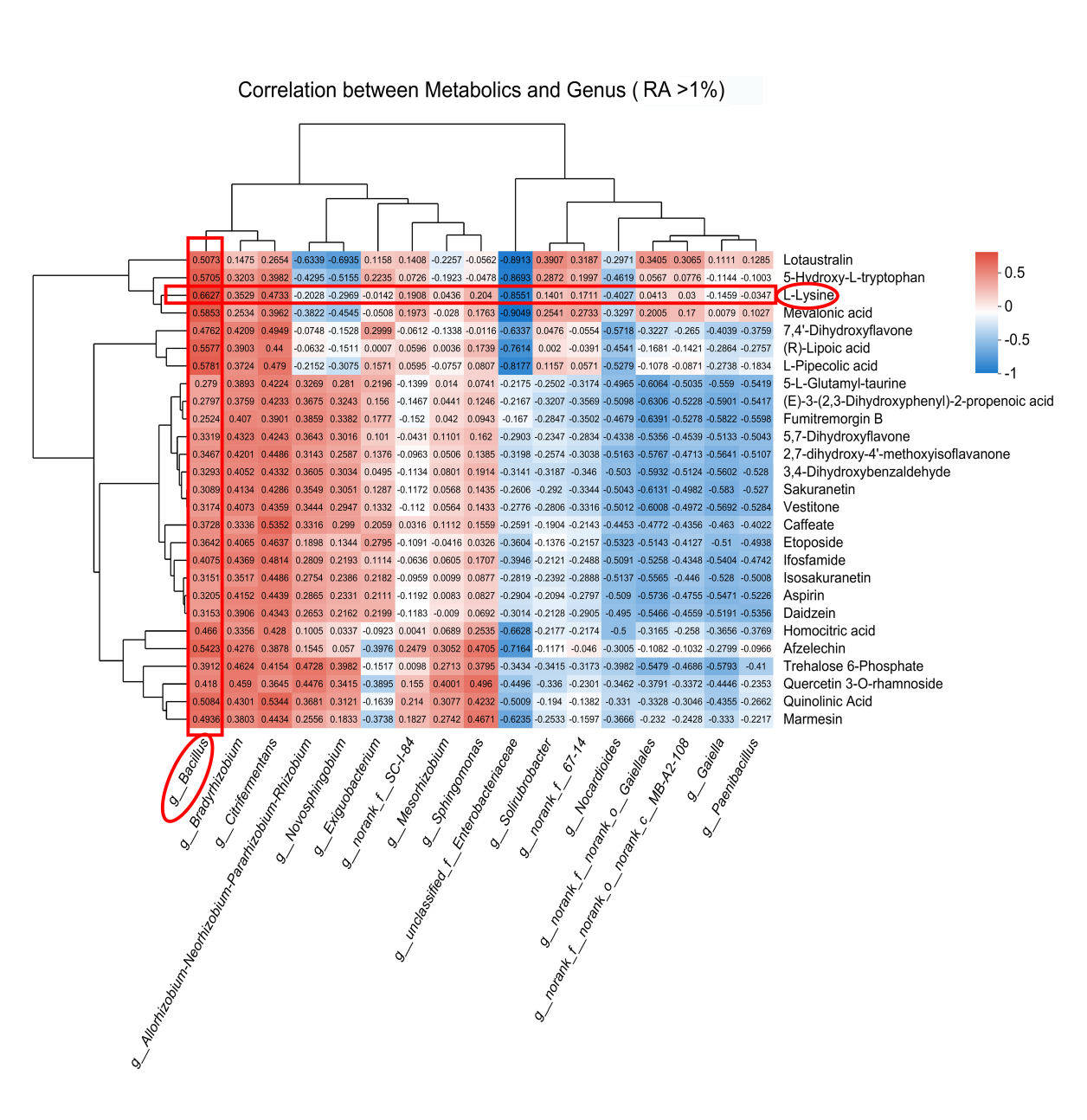
**

**Supplementary Figure 10. Heat maps showing increases in rhizosphere metabolites and rhizosphere bacteria (genus level) of CG935 and M9T337 under *Fpmd* MR5 stress.**


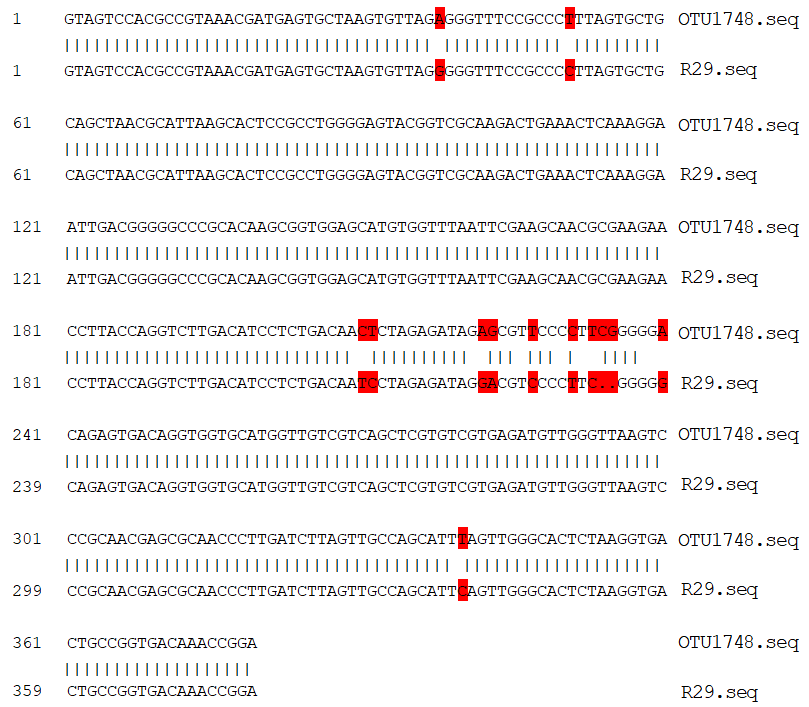


**Supplementary Figure 11. Sequence alignments of the 16S rRNA gene sequence between strain R29 and OTU1748.** Red indicate unmatched nucleotides. Strain R29 showed 96.57% match to the OTU1748.

**
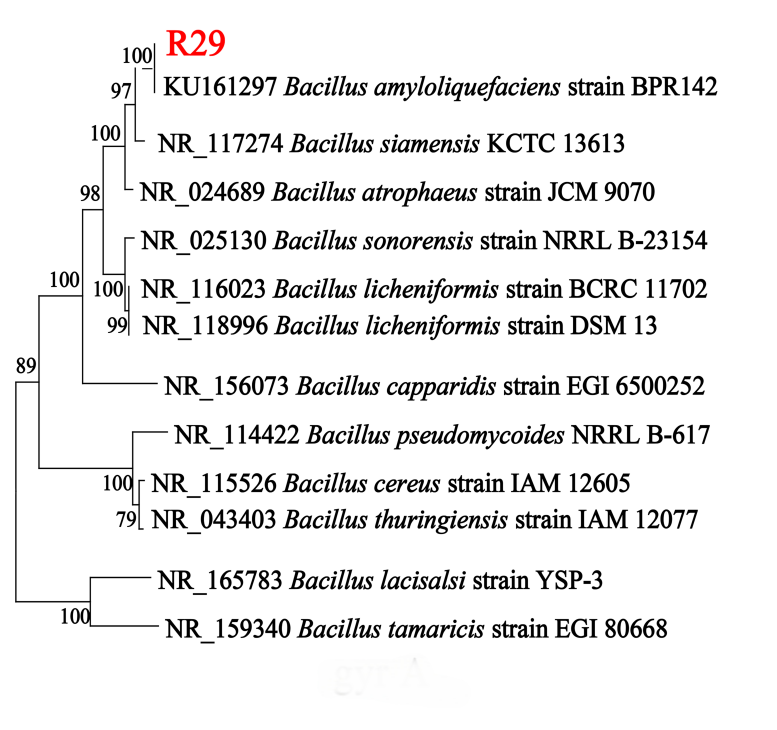
**

**Supplementary Figure 12. Identification of *gyr* A gene sequence of strain R29.**


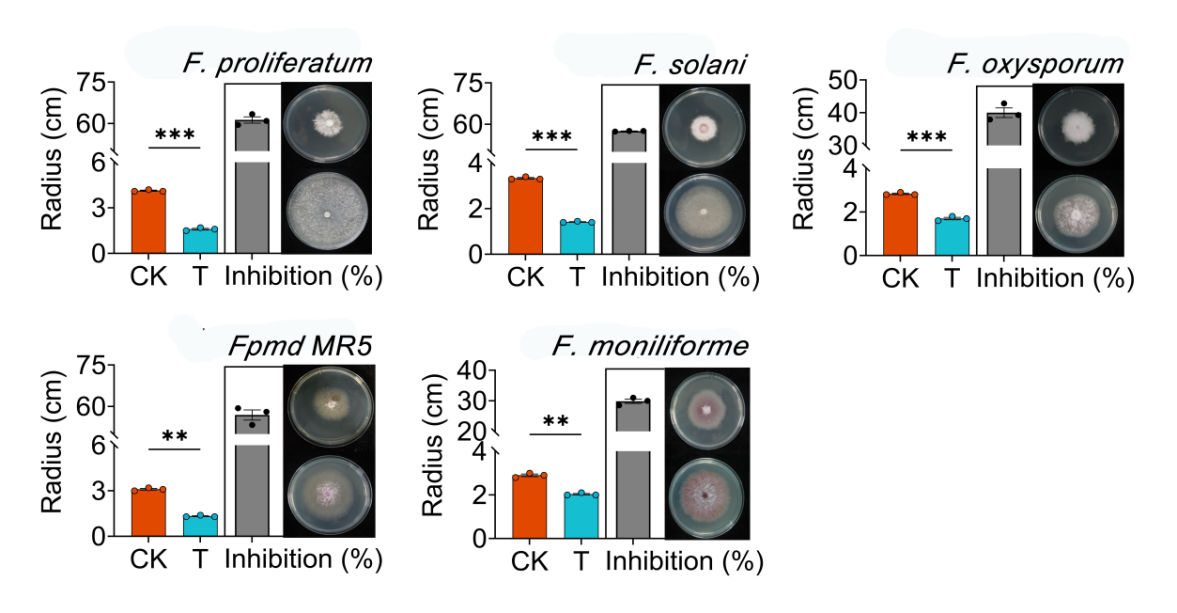


**Supplementary Figure 13.** **Effects of fermentation filtrate of R29 strain on the growth of *F. proliferatum*, *F. solani*, *F. oxysporum*, *Fpmd* MR5, and *F. moniliforme.*** The asterisks on the bars indicate significant differences between the two groups according to a two-tailed Student’s *t*-test (*0.01<P≤0.05, **0.001<P≤0.01, ***P≤0.001) and the mean ± SEM (n=3) for each histogram.

CK: R29 fermentation solution not added to the culture medium, T: R29 fermentation solution added to the culture medium.


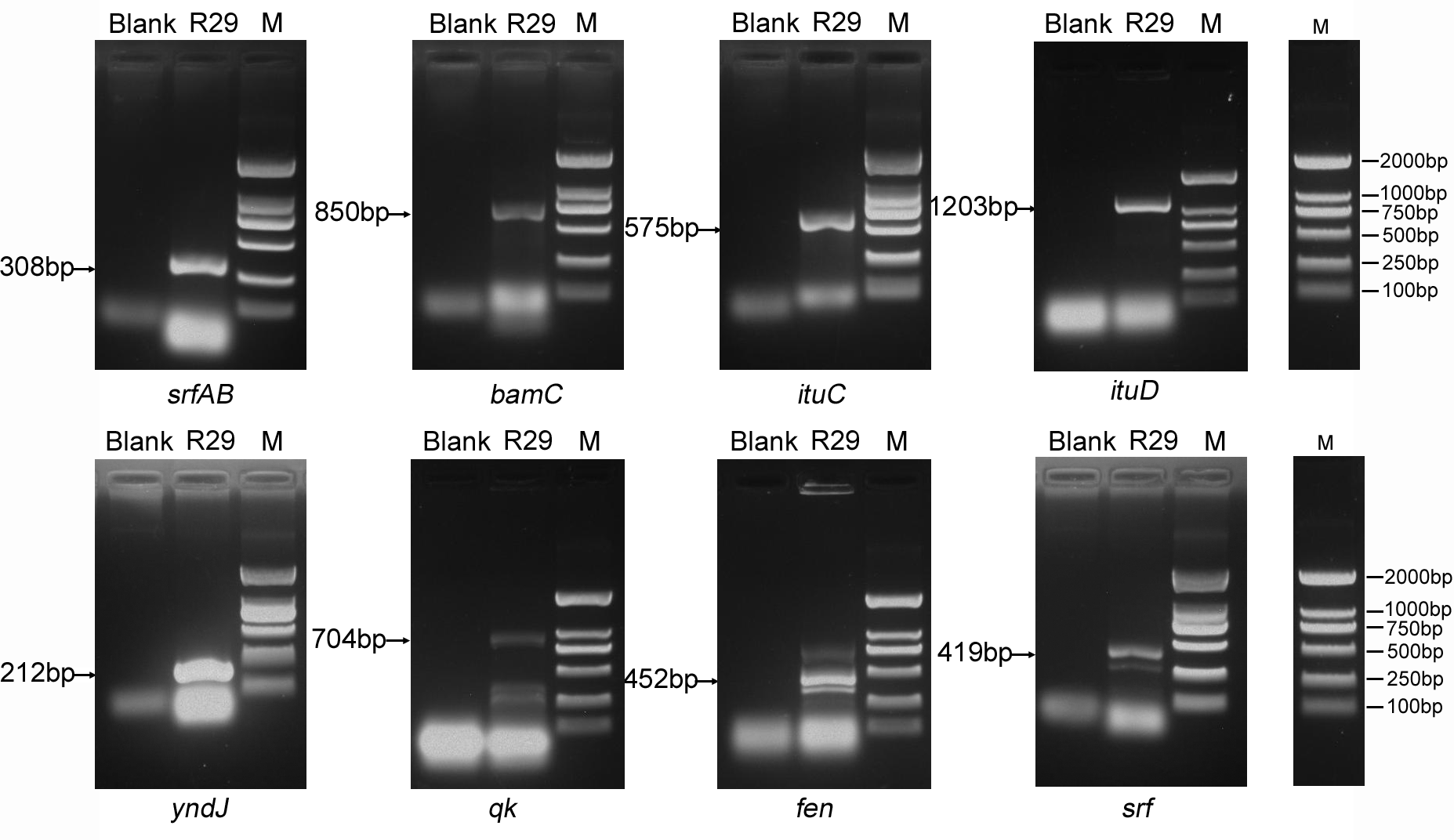


**Supplementary Figure 14. PCR detection of antibiotic synthesis genes in R29 strains.** M: DL2000 Marker.


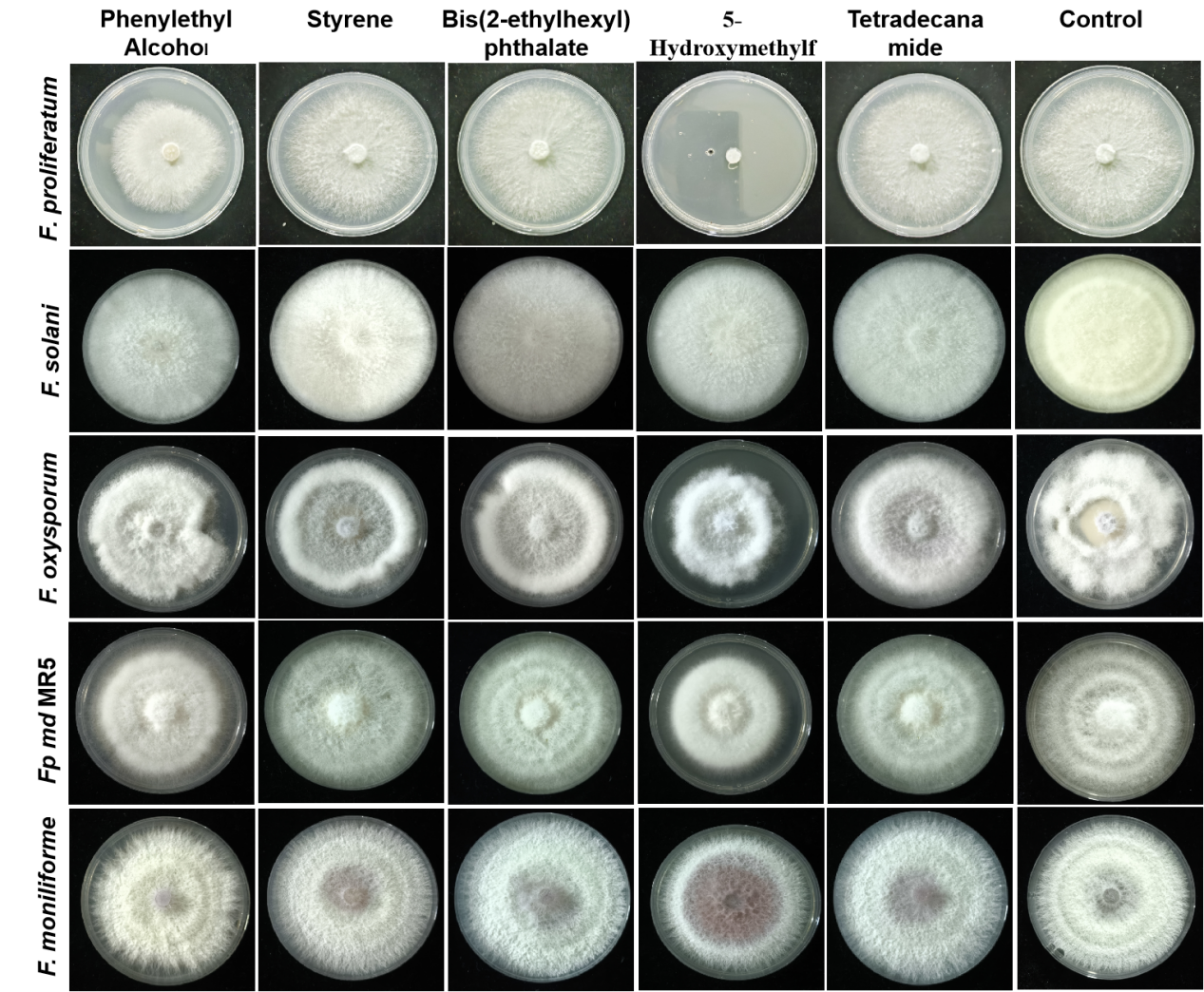


**Supplementary Figure 15. Effects of volatile substances produced by strain R29 on the growth of** ***F. proliferatum*, *F. solani*, *F. oxysporum*, *Fpmd* MR5, and *F. moniliforme.***

**
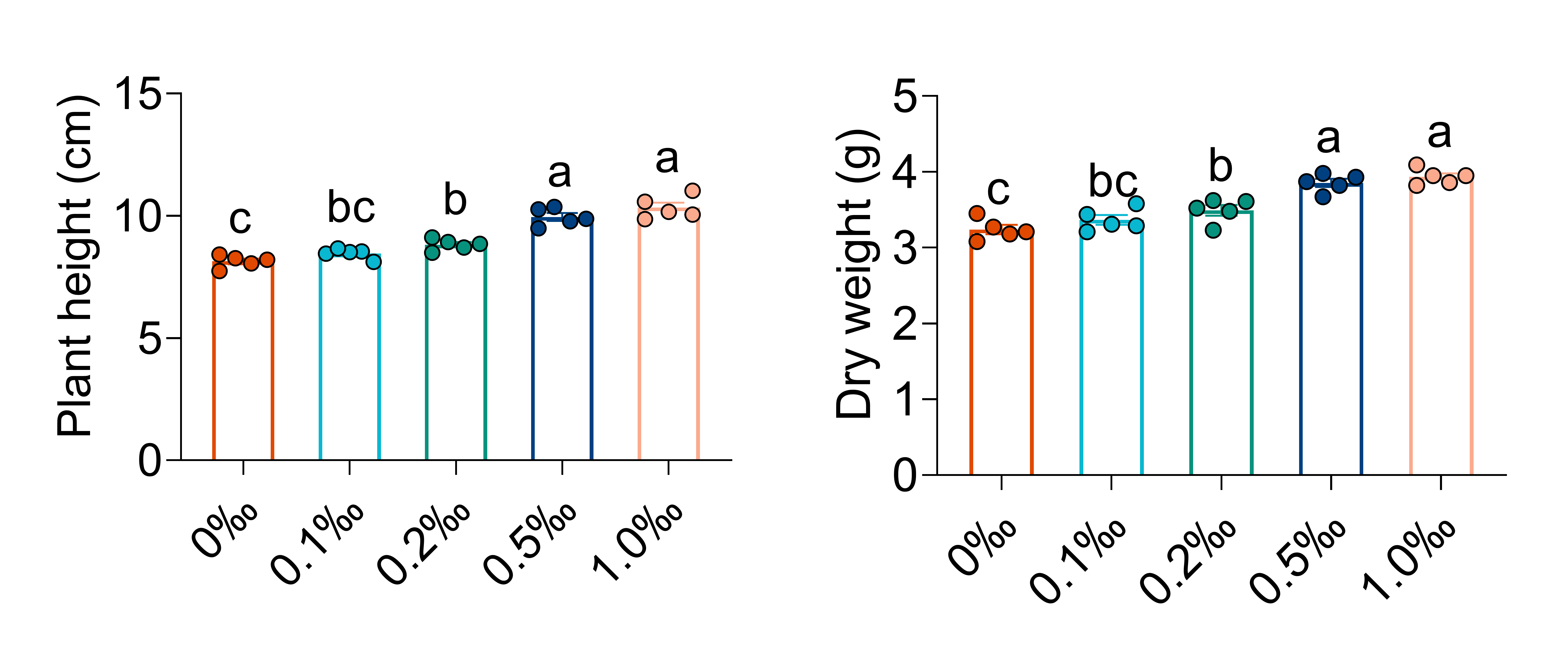
**

**Supplementary Figure 16. Effects of different lysine concentrations on the growth of replanted plants.** The different letters on the bars indicate the significant difference between treatments (P<0.05) by one-way ANOVA. Mean ± SEM for each histogram (n=5).

**
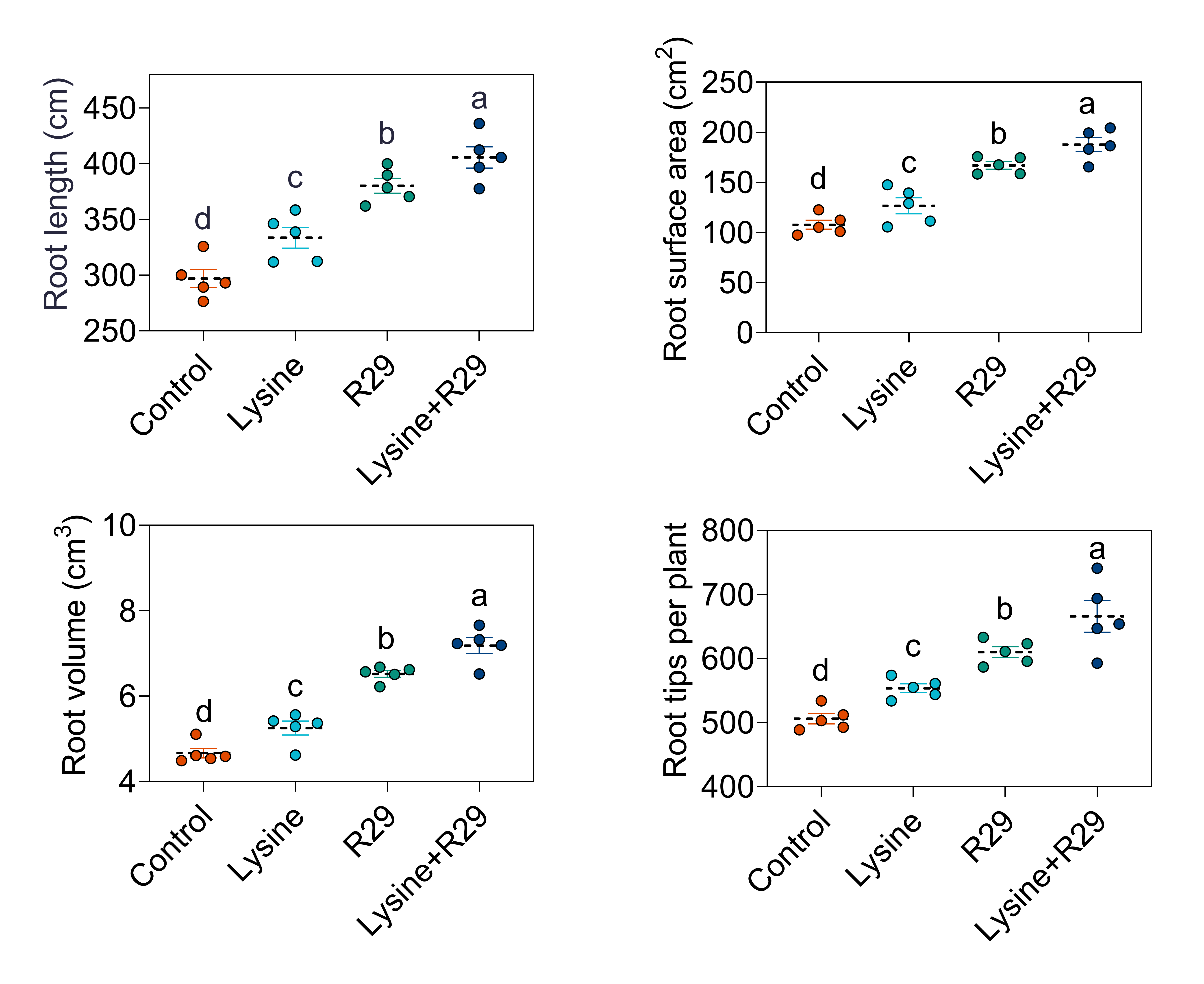
**

**Supplementary Figure 17. Effects of lysine and strain R29 on root length, root surface area, root volume and number of root tips.** The different letters on the bars indicate the significant difference between treatments (P<0.05) by one-way ANOVA.

**
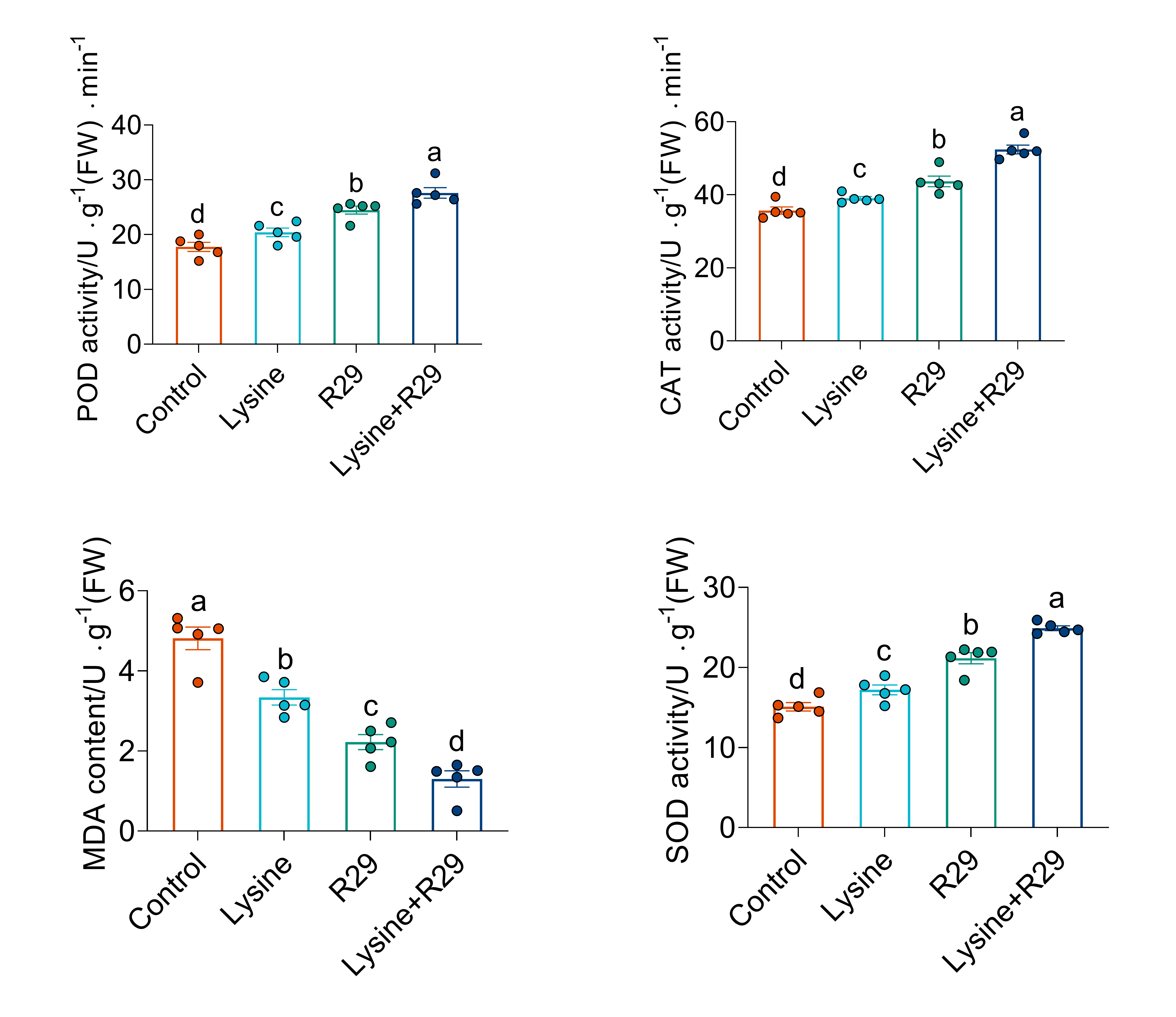
**

**Supplementary Figure 18. Effects of lysine and strain R29 on root protective enzyme activity and malondialdehyde content of replanted M9T337.** The different letters on the bars indicate the significant difference between treatments (P<0.05) by one-way ANOVA. Mean ± SEM for each histogram (n=5).

**
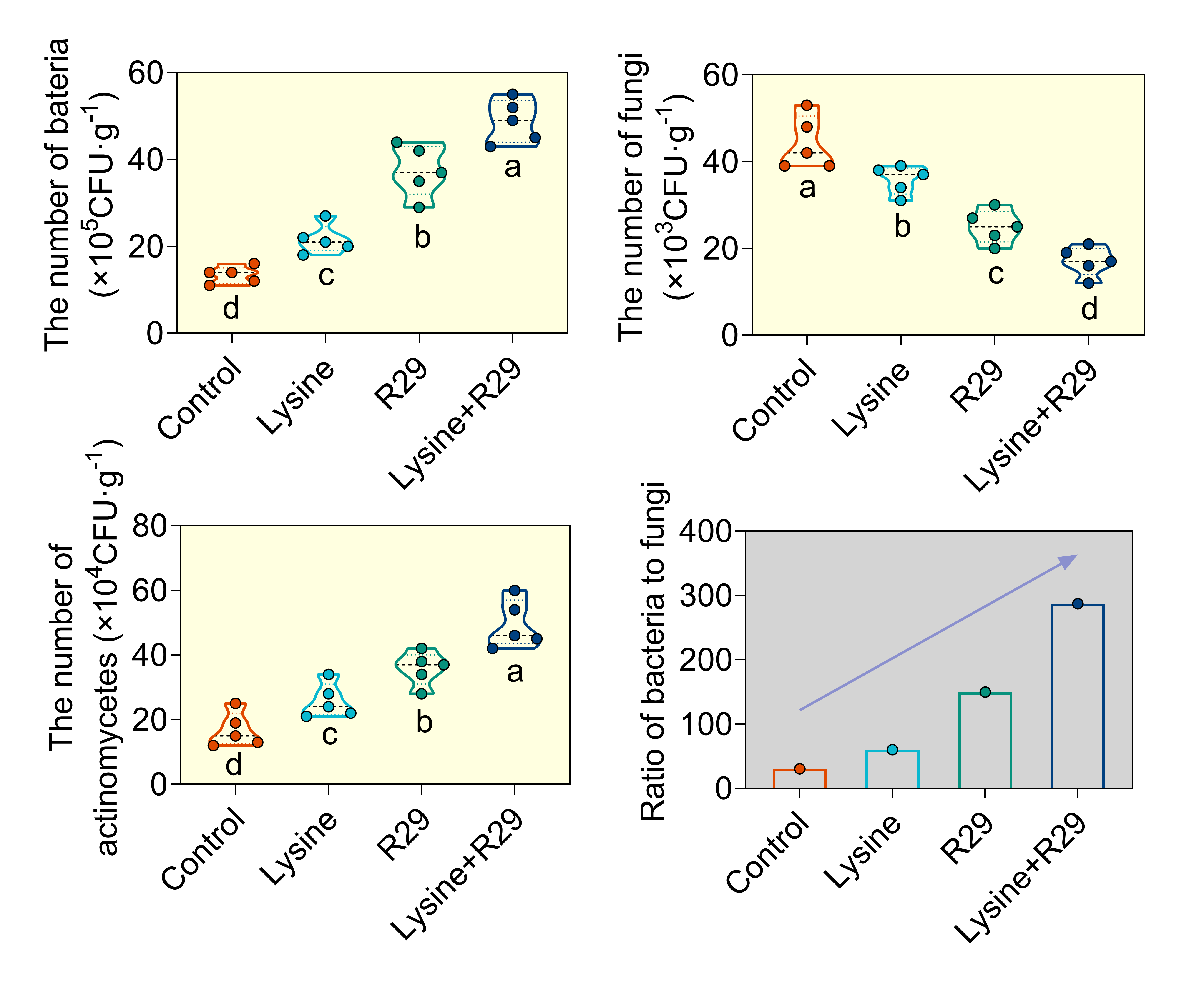
**

**Supplementary Figure 19. Effects of lysine and strain R29 on the number of cultivable microorganisms in the rhizosphere soil of replanted M9T337.** The different letters on the bars indicate the significant difference between treatments (P<0.05) by one-way ANOVA.

**
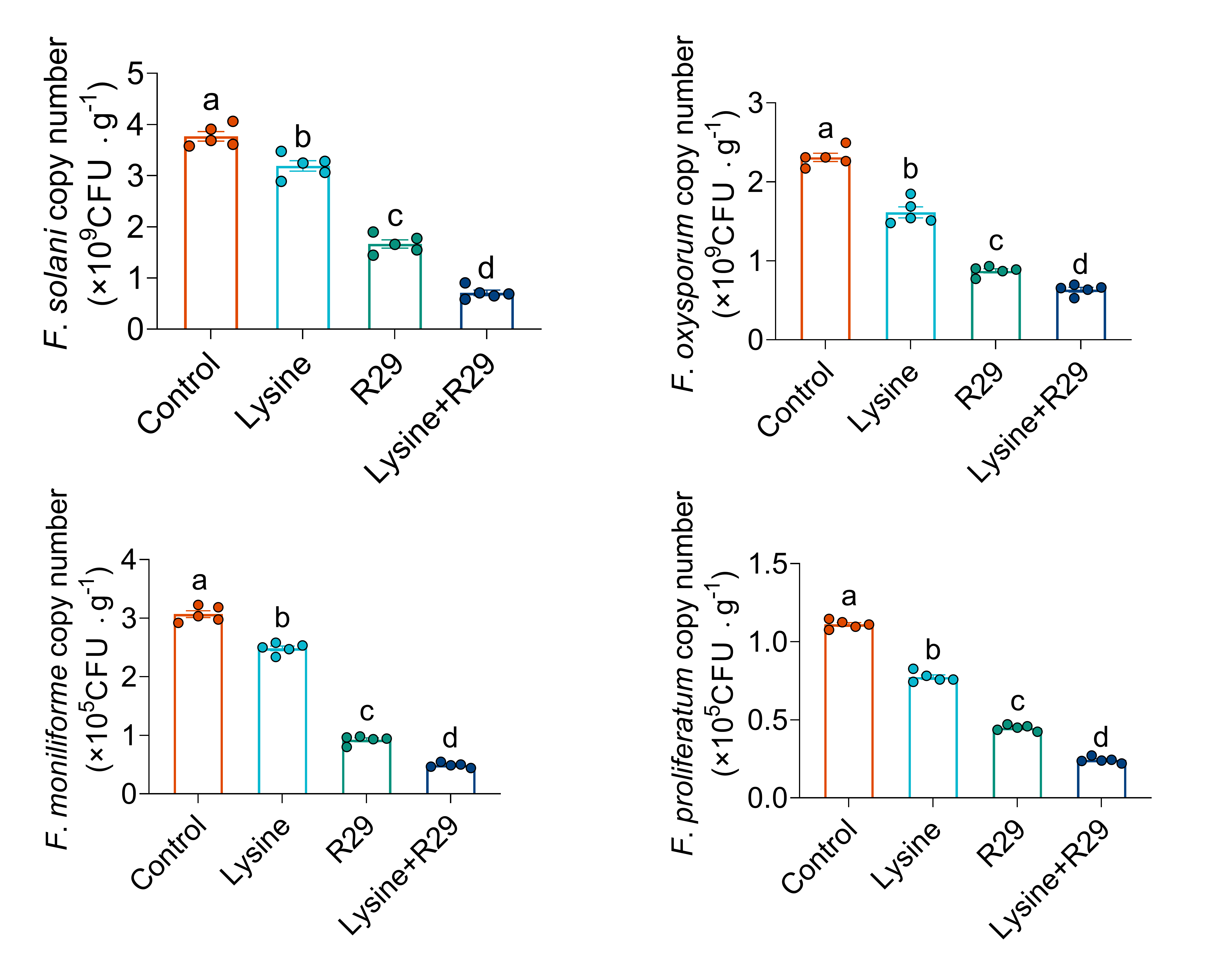
**

**Supplementary Figure 20. Effects of lysine and strain R29 on the quantity of pathogen *Fusarium* in the rhizosphere soil of replanted M9T337.** The different letters on the bars indicate the significant difference between treatments (P<0.05) by one-way ANOVA. Mean ± SEM for each histogram (n=5).

**
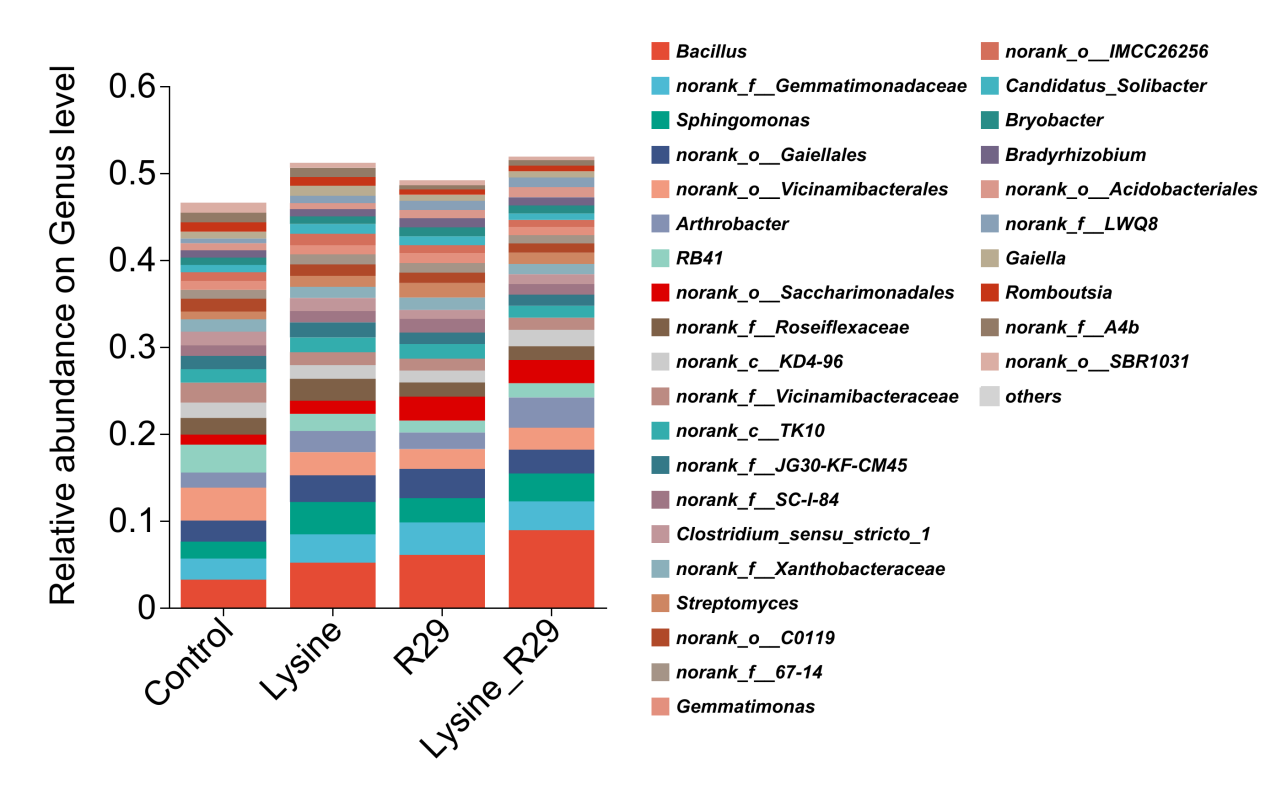
**

**Supplementary Figure 21. Effects of lysine and strain R29 on rhizosphere bacterial community composition (genus level) of replanted M9T337.**


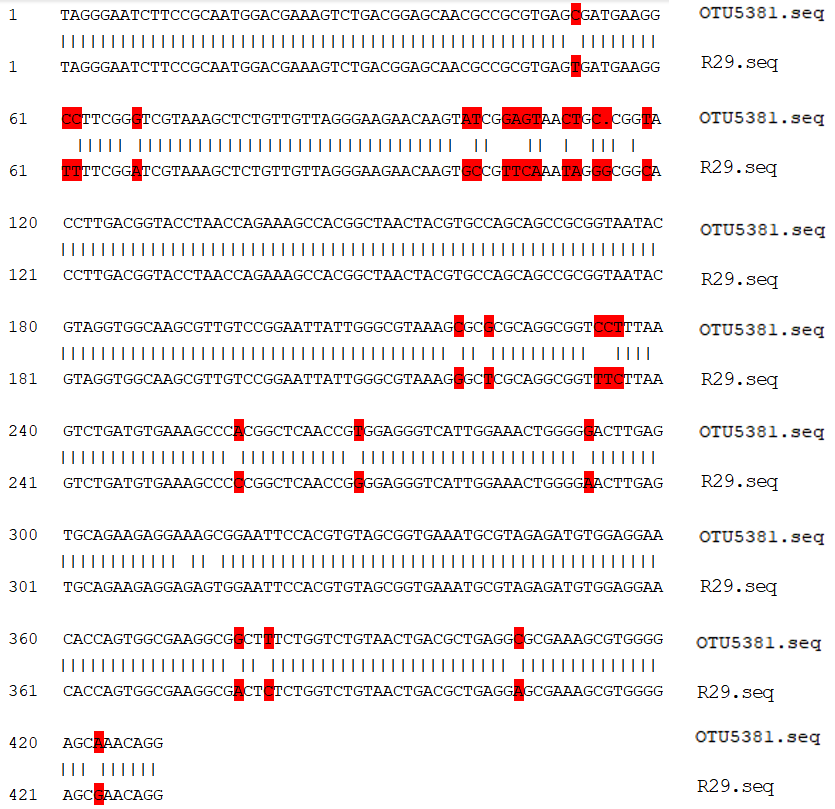


**Supplementary Figure 22. Sequence alignments of the 16S rRNA gene sequence between strain R29 and OTU5381.** Red indicate unmatched nucleotides. Strain R29 showed 93.72% match to the OTU5381.

**
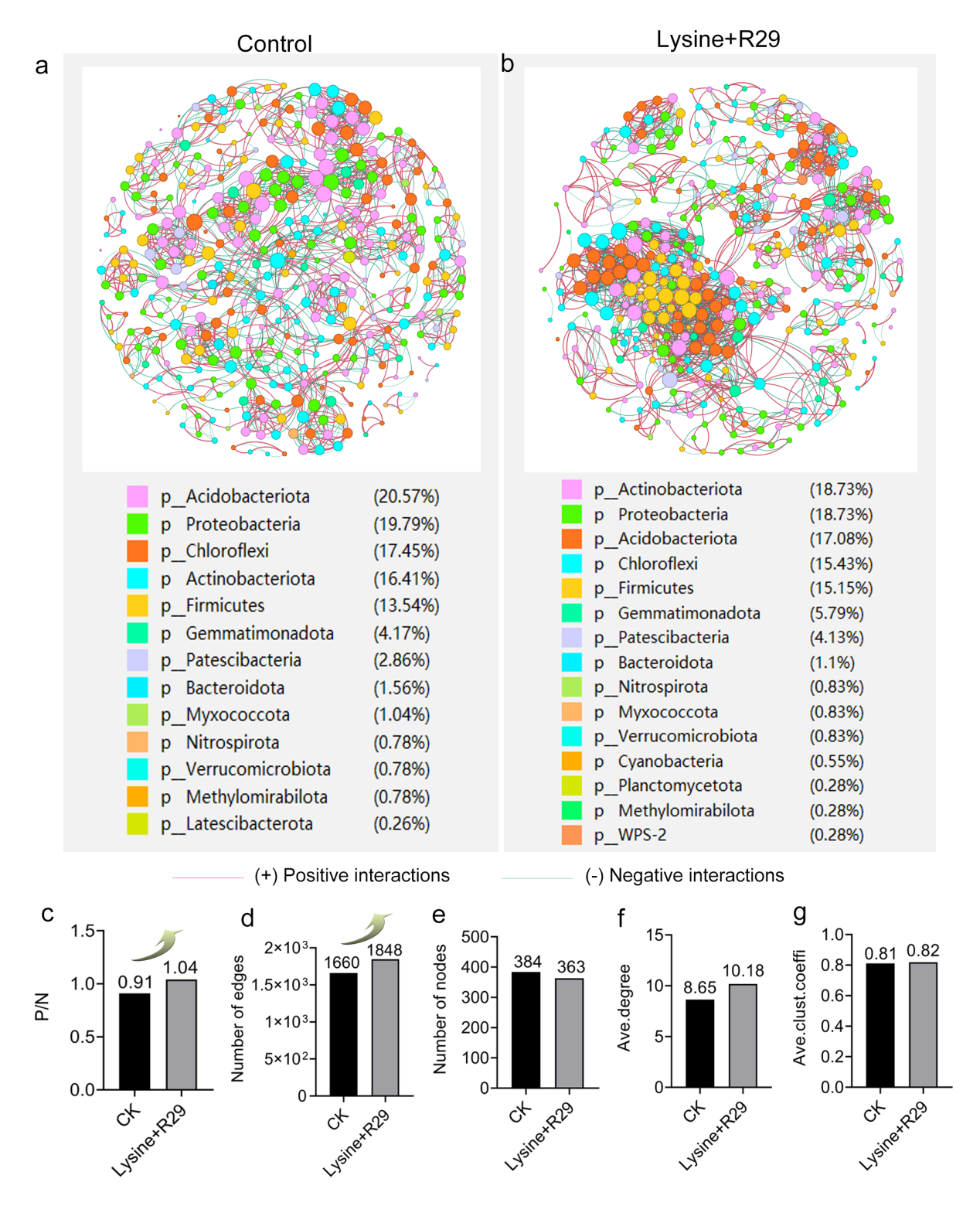
**

**Supplementary Figure 23. Effects of lysine and strain R29 on rhizosphere bacterial community structure-collinear network of replanted M9T337. a** Control bacterial collinearity network; **b** Lysine+strain R29 bacterial collinearity network; **c** Positive and negative correlation ratio of bacterial species; **d** Number of bacterial edges; **e** Number of bacterial nodes; **f** Ave. degree; **g** Ave. cluster. coeffi.


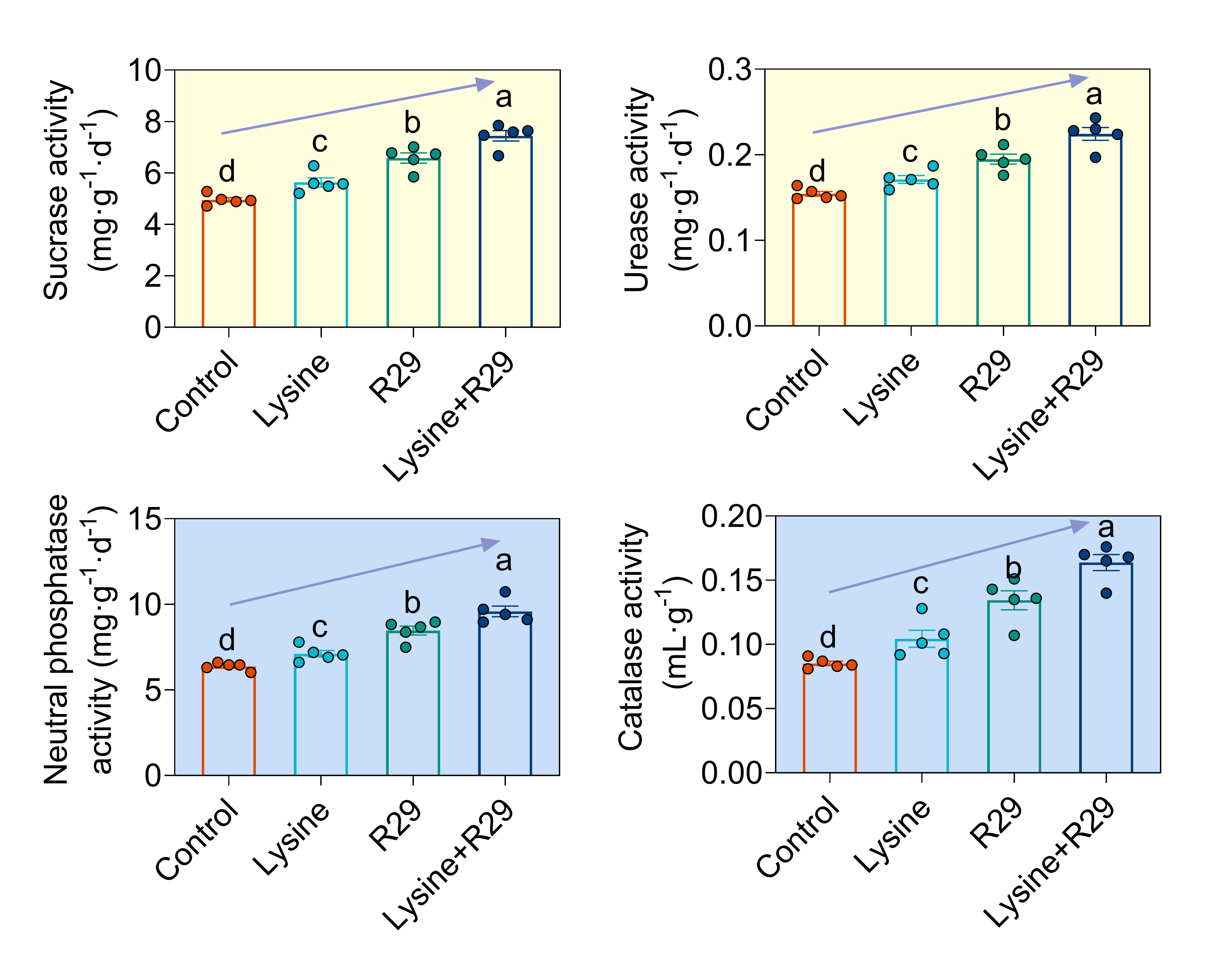


**Supplementary Figure 24. Effects of lysine and strain R29 on rhizosphere soil enzyme activity of replanted M9T337.** The different letters on the bars indicate the significant difference between treatments (P<0.05) by one-way ANOVA. Mean ± SEM for each histogram (n=5).


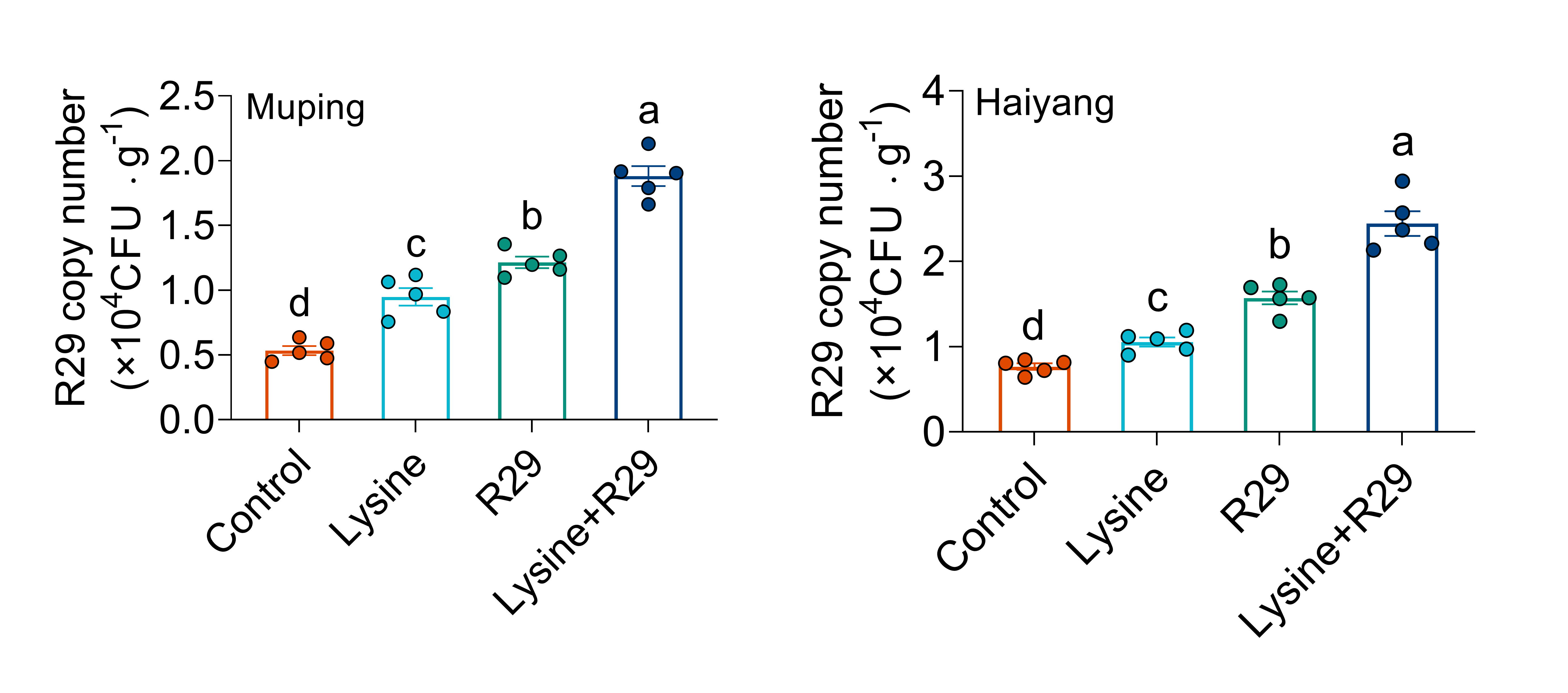


**Supplementary Figure 25. The number of R29 in the rhizosphere of field (2025) plants treated with lysine and strain R29.**

**
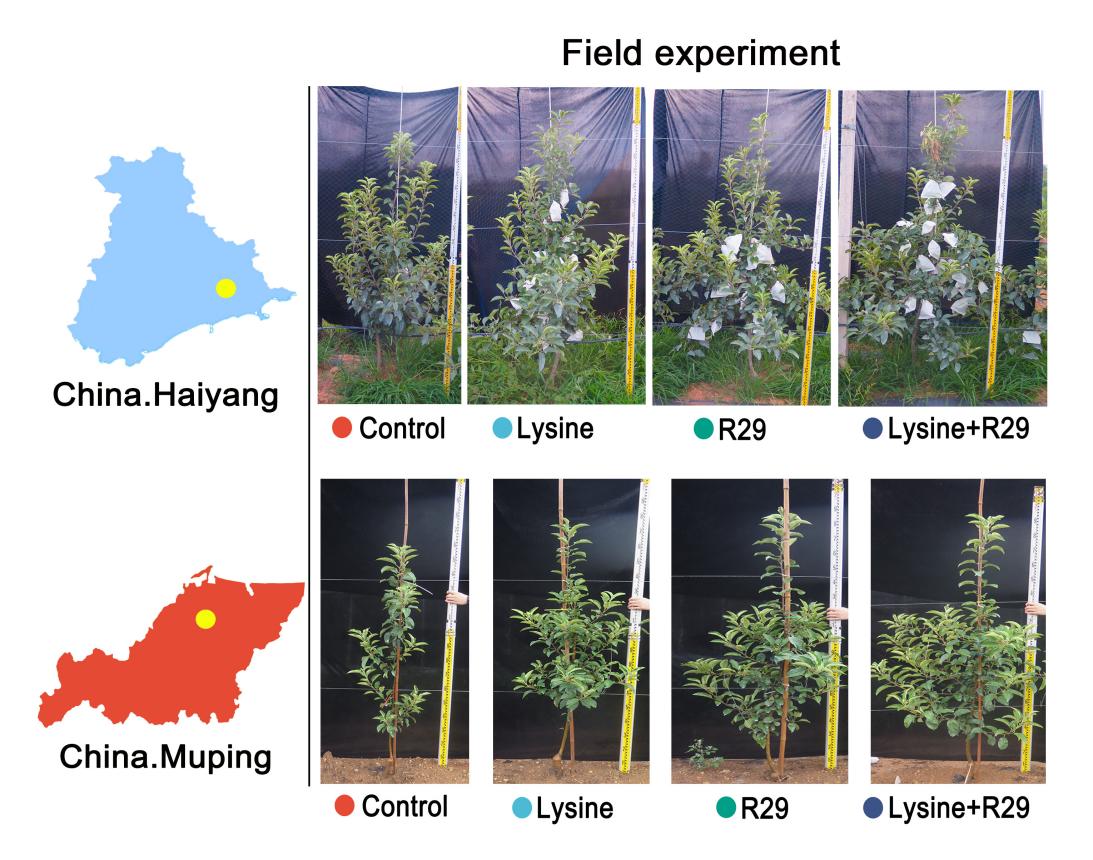
**

**Supplementary Figure 26. Phenotypes of field (2025) plants treated with lysine and strain R29.**

**Supplementary Table 1 Functional analysis of rhizosphere bacterial communities in CG935 and M9T337.**

| KEGG Pathway level 1 | KEGG Pathway level 2 | KEGG Pathway level 3 | Level 3 Description |
| --- | --- | --- | --- |
| Metabolism | Amino acid metabolism | ko00400 | Phenylalanine, tyrosine and tryptophan biosynthesis |
| Metabolism | Amino acid metabolism | ko00380 | Tryptophan metabolism |
| Metabolism | Amino acid metabolism | ko00360 | Phenylalanine metabolism |
| Metabolism | Amino acid metabolism | ko00350 | Tyrosine metabolism |
| Metabolism | Amino acid metabolism | ko00340 | Histidine metabolism |
| Metabolism | Amino acid metabolism | ko00330 | Arginine and proline metabolism |
| Metabolism | Amino acid metabolism | ko00310 | **Lysine degradation** |
| Metabolism | Amino acid metabolism | ko00300 | Lysine biosynthesis |
| Metabolism | Amino acid metabolism | ko00290 | Valine, leucine and isoleucine biosynthesis |
| Metabolism | Amino acid metabolism | ko00280 | Valine, leucine and isoleucine degradation |
| Metabolism | Amino acid metabolism | ko00270 | Cysteine and methionine metabolism |
| Metabolism | Amino acid metabolism | ko00260 | Glycine, serine and threonine metabolism |
| Metabolism | Amino acid metabolism | ko00250 | Alanine, aspartate and glutamate metabolism |

**Supplementary Table 2. Analysis of differential metabolites in amino acid metabolism pathways of CG935 and M9T337.**

| Number | ID | Metabolite |
| --- | --- | --- |
| 1 | pos_3918 | Mevalonic acid |
| 2 | pos_3488 | Afzelechin |
| 3 | pos_3491 | 7,4'-Dihydroxyflavone |
| 4 | pos_3994 | Lotaustralin |
| 5 | pos_1713 | Etoposide |
| 6 | pos_1695 | Quercetin 3-O-rhamnoside |
| 7 | pos_3600 | 5,7-Dihydroxyflavone |
| 8 | pos_3552 | Daidzein |
| 9 | pos_1443 | L-Pipecolic acid |
| 10 | pos_1473 | 5-Hydroxy-L-tryptophan |
| 11 | pos_3550 | Aspirin |
| 12 | pos_3569 | Isosakuranetin |
| 13 | pos_3791 | Marmesin |
| 14 | pos_1649 | 5-L-Glutamyl-taurine |
| 15 | pos_1322 | (R)-Lipoic acid |
| 16 | pos_1403 | L-Lysine |
| 17 | pos_3485 | Sakuranetin |
| 18 | pos_3570 | Caffeate |
| 19 | pos_1298 | Homocitric acid |
| 20 | pos_3455 | 2,7-dihydroxy-4'-methoxyisoflavanone |
| 21 | pos_1738 | (E)-3-(2,3-Dihydroxyphenyl)-2-propenoic acid |
| 22 | pos_701 | Vestitone |
| 23 | pos_3576 | Fumitremorgin B |
| 24 | pos_1434 | Quinolinic Acid |
| 25 | pos_219 | Ifosfamide |
| 26 | pos_3369 | 3,4-Dihydroxybenzaldehyde |
| 27 | pos_3659 | Trehalose 6-Phosphate |

**Supplementary Table 3. Analysis of differential metabolites in amino acid metabolism pathways of CG935 and M9T337.**

| Treatment | KEGG Pathway level 2 | Common differential metabolites |
| --- | --- | --- |
| GZ_RS VS GZRS  GZ_RS VS MZ_RS | Metabolism of other amino acids | Lotaustralin |
| GZ_RS VS GZRS  GZ_RS VS MZ_RS | Amino acid metabolism | L-Pipecolic acid |
| GZ_RS VS GZRS  GZ_RS VS MZ_RS | Amino acid metabolism | 5-Hydroxy-L-tryptophan |
| GZ_RS VS GZRS  GZ_RS VS MZ_RS | Amino acid metabolism | **L-Lysine** |
| GZ_RS VS GZRS  GZ_RS VS MZ_RS | Amino acid metabolism | Homocitric acid |
| GZ_RS VS GZRS  GZ_RS VS MZ_RS | Amino acid metabolism | （(E)-3-(2,3-Dihydroxyphenyl)-2-propenoic acid |
| GZ_RS VS GZRS  GZ_RS VS MZ_RS | Amino acid metabolism | Quinolinic Acid |

**Supplementary Table 4. Physiological, biochemical properties and function determination of strain R29.**

| Experimental names | Results and phenotype | | Experimental names | Results and phenotype | |
| --- | --- | --- | --- | --- | --- |
| Starch hydrolysis | + | 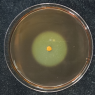 | Proteolysis | + | 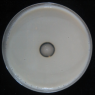 |
| Inorganic phosphorus | - | 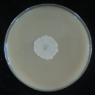 | Nitrogen fixation | - | 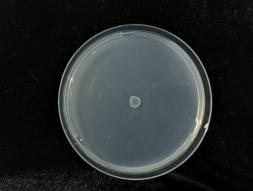 |
| Phloridzin degradation | - | 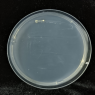 | Siderophore production | - | 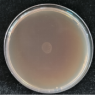 |
| Siderophoreproduction | + | 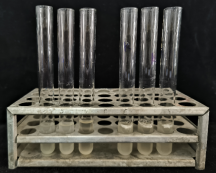 | Cellulolysis | - | 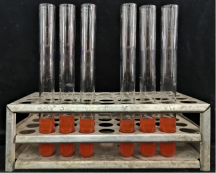 |
| Methyl red | + | 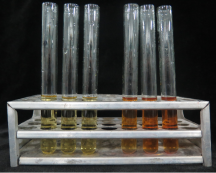 | V-P test | + | 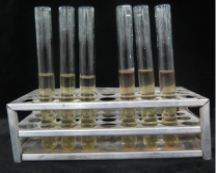 |
| Ammonia production | + | 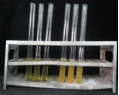 | Gelatin liquefaction | + | 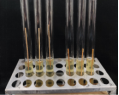 |
| Indole production | - | 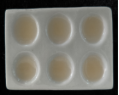 | Nitrate reduction | + | 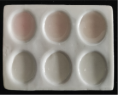 |

**Supplementary Table 5. Effects of fermentation filtrate of strain R29 on the spore germination of *F. proliferatum*, *F. solani*, *F. oxysporum*, *Fpmd* MR5, and *F. moniliforme.***

| Treatment | *F. proliferatum* | | *F. moniliforme* | | *F. oxysporum* | | *F. solani* | | *Fpmd* MR5 | |
| --- | --- | --- | --- | --- | --- | --- | --- | --- | --- | --- |
|  | Germination rate（%） | Inhibition rate（%） | Germination rate（%） | Inhibition rate（%） | Germination rate（%） | Inhibition rate（%） | Germination rate（%） | Inhibition rate（%） | Germination rate（%） | Inhibition rate（%） |
| CK | 96.02±0.78*** | - | 89.38±0.73*** | - | 91.26±1.42*** | - | 81.26±1.99** | - | 91.41±0.87** | - |
| T | 51.89±0.58 | 44.13 | 47.96±0.83 | 41.42 | 47.19±0.91 | 44.07 | 49.30±0.79 | 31.96 | 48.76±1.56 | 42.65 |

The asterisk indicate significant differences between the two groups according to a two-tailed Student’s *t*-test (*0.01<P≤0.05, **0.001<P≤0.01, ***P≤0.001), and all data are mean±SEM (n=3).

**Supplementary Table 6. Composition of fermentation filtrate of strain R29 was determined by GC-MS.**

| Number | Retention time/min | Peak area/% | Ingredient name |
| --- | --- | --- | --- |
| 1 | 4.975 | 0.58 | Benzene, 1,3-dimethyl- |
| 2 | 5.202 | 0.43 | Butanoic acid, 3-methyl- |
| 3 | 5.31 | 0.52 | Styrene |
| 4 | 5.48 | 1.76 | Butanoic acid, 2-methyl- |
| 5 | 6.569 | 0.3 | Ethanol, 2,2'-oxybis- |
| 6 | 7.528 | 0.11 | 1,2-Cyclopentanedione, 3-methyl- |
| 7 | 8.918 | 0.12 | Phenylethyl Alcohol |
| 8 | 11.668 | 0.19 | 5H-1-Pyrindine |
| 9 | 14.425 | 0.26 | 2,4-Di-tert-butylphenol |
| 10 | 17.259 | 2.34 | 1,4-diazabicyclo[4.3.0]nonan-2,5-dione, 3-methyl |
| 11 | 17.925 | 1.9 | (3S,6S)-3-Butyl-6-methylpiperazine-2,5-dione |
| 12 | 18.152 | 0.59 | 5-Hydroxymethylfurfural |
| 13 | 18.408 | 5.7 | 3-Isopropyl-2,5-piperazine-dione |
| 14 | 18.513 | 5.32 | Cyclo(L-prolyl-L-valine) |
| 15 | 18.704 | 3.97 | Cyclo(L-prolyl-L-valine) |
| 16 | 18.9 | 0.62 | 3,6-Diisopropylpiperazin-2,5-dione |
| 17 | 18.947 | 0.29 | 3,6-Diisopropylpiperazin-2,5-dione |
| 18 | 19.025 | 0.13 | Pyrimidine, 2,5-dimethoxy- |
| 19 | 19.357 | 6.98 | Pyrrolo[1,2-a]pyrazine-1,4-dione, hexahydro-3-(2-methylpropyl)- |
| 20 | 19.572 | 8.74 | Phthalic acid, 5-methylhex-2-yl butyl ester |
| 21 | 19.67 | 4.93 | Pyrrolo[1,2-a]pyrazine-1,4-dione, hexahydro-3-(2-methylpropyl)- |
| 22 | 19.775 | 0.09 | 5,5-Diethylheptadecane |
| 23 | 19.888 | 0.4 | 4(3H)-Pyrimidinone, 3-ethyl-2,6-dimethyl- |
| 24 | 20.02 | 0.39 | 1H-Indole-3-acetic acid, methyl ester |
| 25 | 20.121 | 0.34 | Tetradecanamide |
| 26 | 20.275 | 0.34 | 1-Hexacosene |
| 27 | 20.41 | 0.58 | Trichloroacetic acid, hexadecyl ester |
| 28 | 20.8 | 0.22 | Oxacycloheptadec-8-en-2-one, (8Z)- |
| 29 | 20.963 | 0.12 | 4,5-Diphenylocta-1,7-diene(meso) |
| 30 | 21.111 | 0.18 | Hexadecanamide |
| 31 | 21.217 | 0.14 | Sulfurous acid, octadecyl 2-propyl ester |
| 32 | 21.322 | 0.41 | 2,5-Piperazinedione, 3-methyl-6-(phenylmethyl)- |
| 33 | 21.439 | 0.86 | 2,5-Piperazinedione, 3,6-bis(2-methylpropyl)- |
| 34 | 21.573 | 1.37 | 2,5-Piperazinedione, 3-(phenylmethyl)- |
| 35 | 21.716 | 3.59 | Pyrrolo[1,2-a]pyrazine-1,4-dione, hexahydro-3-(2-methylpropyl)- |
| 36 | 21.808 | 0.52 | 6,7-Epoxyoctadecanoic acid methyl ester |
| 37 | 21.899 | 1.47 | Pyrrolo[1,2-a]pyrazine-1,4-dione, hexahydro-3-(2-methylpropyl)- |
| 38 | 21.992 | 0.13 | 2,5-Piperazinedione, 3-benzyl-6-isopropyl- |
| 39 | 22.266 | 0.28 | 9-Octadecenamide, (Z)- |
| 40 | 22.501 | 7.27 | Pyrrolo[1,2-a]pyrazine-1,4-dione, hexahydro-3-(phenylmethyl)- |
| 41 | 22.704 | 10.1 | Pyrrolo[1,2-a]pyrazine-1,4-dione, hexahydro-3-(phenylmethyl)- |
| 42 | 22.898 | 0.47 | Cyclohexane, 1,3,5-triphenyl- |
| 43 | 23.183 | 0.12 | Cyclononasiloxane, octadecamethyl- |
| 44 | 23.321 | 3.67 | Bis(2-ethylhexyl) phthalate |
| 45 | 23.425 | 0.16 | 9-Octadecenamide, (Z)- |
| 46 | 23.466 | 0.2 | Behenyl chloride |
| 47 | 23.541 | 0.13 | (2,3-Diphenylcyclopropyl)methyl phenyl sulfoxide, trans- |
| 48 | 23.732 | 0.42 | Benzamide, 2,5-difluoro-N-benzyl-N-hexyl- |
| 49 | 23.883 | 0.1 | Cyclononasiloxane, octadecamethyl- |
| 50 | 23.96 | 0.47 | L-Prolinamide, 5-oxo-L-prolyl-L-phenylalanyl-4-hydroxy- |
| 51 | 24.075 | 0.2 | Decane, 1-bromo-2-methyl- |
| 52 | 24.135 | 0.15 | 2,5-Piperazinedione, 2-(2-methylthio)ethyl-6-benzyl- |
| 53 | 24.522 | 7.3 | 13-Docosenamide, (Z)- |
| 54 | 24.655 | 6.05 | Benzenemethanol, .alpha.-(3-methyl-2-butenyl)- |
| 55 | 24.808 | 4.41 | Formic acid, (2-fluoro-5-nitrophenyl)methyl ester |
| 56 | 24.925 | 0.18 | 2,5-Piperazinedione, 3,6-bis(phenylmethyl)- |
| 57 | 25.041 | 0.1 | Pentatriacontane |
| 58 | 26.358 | 0.69 | Benzenepropanoic acid, 3,5-bis(1,1-dimethylethyl)-4-hydroxy-, octadecyl ester |
| 59 | 26.545 | 0.35 | Tris(2,4-di-tert-butylphenyl) phosphate |
| 60 | 26.884 | 0.23 | Tryptophan |

**Supplementary Table 7. Sequence of antibiotic synthesis gene primers.**

| Antibiotics | Target gene | Code | Sequences (5’-3’) | Amplicon size (bp) |
| --- | --- | --- | --- | --- |
| Fengycin | *fen* | Af2-F/Tf1-R | GAATAYMTCGGMCGTMTKGA  GCTTTWADKGAATSBCCGCC | 452 |
| Surfactin | *srf* | As1-F/Ts2-R | CGCGGMTACCGVATYGAGC  ATBCCTTTBTWDGAATGTCCGCC | 419 |
| Iturin A | *ituD* | ituD-F/ituD-R | ATGAACAATCTTGCCTTTTTA  TTATTTTAA AAT CCGCAATT | 1203 |
| Subtilosin | *sboA* | Sbo1F/Sbo1R | TCGGTTTGTAAACTTCAACTGC  GTCCACTAGACAAGCGGCTC | 334 |
| Yndj | *yndJ* | 147F/147R | CAGAGCGACAGCAATCACAT  TGAATTTCGGTCCGCTTATC | 212 |
| Subtilisin | *qk* | Qk1F/Qk1R | CTTAAACGTCAGAGGCGGAG  ATTGTGCAGCTGCTTGTACG | 704 |
| Surfactin | *srfAB* | 110F/110R | GTTCTCGCAGTCCAGCAGAAG  GCCGAGCGTATCCGTACCGAG | 308 |
| Bacillomycin | *bamC* | bamC2F/bamC2R | CTGGAAGAGATGCCGCTTAC  AAGAGTGCGTTTTCTTCGGA | 850 |
| Iturin | *ituC* | ITUCF1/ITUCR3 | TTCACTTTTGATCTGGCGAT  CGTCCGGTACATTTTCAC | 575 |
| Fengycin | *fenB* | FenB1F/FenB1R | TACCAATCGCAATGTCGTGT  CTTCGATTTCTAACAGCCGC | 767 |

**Supplementary Table 8. Primer, reaction system, and thermal program information of four pathogenic *Fusarium* species.**

| Strain | Primer Sequence | Reaction system | Volumes （uL） | Thermal profile |
| --- | --- | --- | --- | --- |
| *F. oxysporum* | F: 5′-CATACCACTTGTTGTCTCGGC- 3′  R: 5′-GAACGCGAATTAACGCGAGTC- 3′ | SYBR Premix Ex Taq II  Forward primer  Reverse primer  DNA  ddH_2_O | 12.5  1  1  1.5  9 | 95^o^C-30s  95^o^C-5s  60^o^C-30s  40 cycles |
| *F. proliferatum* | F: 5′- GATCGGCGAGCCCTTGCGGCAAG-3′  R: 5′-CGCCGCGTACCAGTTGCGAGGGT- 3′ | SYBR Premix Ex Taq II  Forward primer  Reverse primer  DNA  ddH_2_O | 12.5  1  1  1.5  9 | 95^o^C-30s  95^o^C-5s  65^o^C-30s  40 cycles |
| *F. solani* | F: 5′-CGAGTTATACAACTCATCAACC- 3′  R: 5′-GGCCTGAGGGTTGTAATG- 3′ | SYBR Premix Ex Taq II  Forward primer  Reverse primer  DNA  ddH_2_O | 12.5  1  1  1.5  9 | 95^o^C-30s  95^o^C-5s  55^o^C-30s  40 cycles |
| *F. moniliforme* | F: 5′-GACTCGCGAGTCAAATCGCGT- 3′  R: 5′-GGGGTTTAACGGCGTGGCC- 3′ | SYBR Premix Ex Taq II  Forward primer  Reverse primer  DNA  ddH_2_O | 12.5  1  1  1.5  9 | 95^o^C-30s  95^o^C-5s  60^o^C-30s  40 cycles |

**Supplementary Table 9. Culture medium composition.**

| BPM liquid medium | | Chemotactie buffer | | MSM medium | |
| --- | --- | --- | --- | --- | --- |
| Peptone | 10g | KH_2_PO_4_ | 0.53g | (NH_4_)_2_SO_4_ | 1g |
| Beef paste | 3g | KHPO_4_·3H_2_O | 1.392g | Na_2_HPO_4_·3H_2_O | 7g |
| NaCl | 5g | EDTA | 20 μmol·L^-1^ | KH_2_PO_4_ | 3g |
| pH | 7.2 | pH | 7.0-7.2 | MgSO_4_ | 0.1g |
|  |  |  |  | NaCl | 0.5g |
|  |  |  |  | pH | 7.0-7.2 |
|  |  |  |  | Agar | 2g |
| H_2_O | to 1000 mL | H_2_O | to 1000 mL | H_2_O | to 1000 mL |
